# Prophylactic potential of cytolethal distending toxin B (CdtB) subunit of typhoid toxin against Typhoid fever

**DOI:** 10.1038/s41598-019-54690-1

**Published:** 2019-12-05

**Authors:** Reena Thakur, Preeti Pathania, Navneet Kaur, Vattan Joshi, Kanthi Kiran Kondepudi, Raman Chander Suri, Praveen Rishi

**Affiliations:** 1 Department of Microbiology, Panjab University, Chandigarh, India; 2 National Agri-Food Biotechnology Institute, Mohali, India; 3 Indian Institute of Technology Ropar, Rupnagar, Punjab India

**Keywords:** Protein vaccines, Pathogens

## Abstract

Typhoid fever caused by *Salmonella enterica* serovar Typhi (*S*.Typhi) continues to be a major problem, especially in developing countries. Due to the rapid emergence of multi-drug-resistant (MDR) strains, which limits the efficacy of conventional antibiotics as well as problems associated with the existing vaccines, efforts are being made to develop effective prophylactic agents. CdtB subunit of typhoid toxin was selected for assessing its vaccine potential due to its high conservation throughout the Typhi strains. *In-vitro* assessment of DNase activity of cloned and purified CdtB protein showed a significant decrease in the band intensity of DNA. The measure of metabolic activity and morphological alterations assessed using different cell lines in the presence of CdtB protein showed no significant signs of toxicity. These observations were further strengthened by cell cycle analysis, assessed by flow cytometry. Keeping these observations in mind, the immunoprotective potential of CdtB was assessed using *S*.Typhi induced mouse peritonitis model. A significant titer of IgG antibodies (>128000) against CdtB protein was recorded in the immunized mice by enzyme-linked immunosorbent assay (ELISA), which was also validated by immunoblotting. Active immunization with the protein protected 75% mice against a lethal dose of *S*.Typhi Ty2. The data indicated a significant (up to 5 log) reduction in the bacterial load in the spleen and liver of immunized-infected mice compared to control (unimmunized-infected) mice which might have resulted in the modulation of histoarchitecture of spleen and liver and the levels of cytokines (IL-6, TNF-α and IL-10) production; thereby indicating the effectiveness of the subunit. The observations deduced from the study give the proof of concept of immunogenic potential of protein. However, further studies involving the immunoreactivity of CdtB with the statistically significant number of sera samples obtained from the human patients would be helpful in establishing the relevance of CdtB protein in humans and for making the strategies to develop it as an effective vaccine candidate.

## Introduction

Typhoid fever caused by *Salmonella enterica* serovar Typhi is characterized as a severe systemic febrile illness that accounts for approximately 600,000 deaths annually around the world^1,2^. *S*.Typhi is a Gram-negative, facultative anaerobic bacilli that belong to family *Enterobacteriaceae*. It usually spreads through contaminated food and water sources and is endemic in developing countries particularly in the Asian continent where public health measures are not up to the standard^3–5^. Antibiotics such as amoxicillin, chloramphenicol, and co-trimoxazole have been the mainstay of the treatment against *S*.Typhi. However, due to increase in the emergence of resistant strains these conventional antibiotics are becoming ineffective^6^. Recently in 2017, WHO has announced the list of antibiotic-resistant priority pathogens and mentioned *S*. Typhi as one of them^7^. Hence, to avoid the emergence of resistant variants of this organism; thus reducing the impact of infectious disease especially in endemic areas novel biotherapeutic or prophylactic approaches are direly needed.

In context to prophylactic measures, different types of vaccines against typhoid fever starting from whole-cell vaccines to subunit and to multivalent conjugated vaccines have been tried and tested to prove their efficacy for providing protection. Earlier inactivated (killed) and live whole-cell vaccines were in use^8^. However, due to the high incidence of associated adverse (systemic and local) reactions, short-term immunity provided by the killed vaccine; and requirement of multi-dose regimen, unsuitability for immunocompromised persons, and danger of reverting back to a virulent form of live attenuated whole-cell vaccine had rendered them unsuitable for the use^9^. Later on, with the advancement in the knowledge of important *Salmonella* antigens playing role in its pathogenesis has shifted focus towards the development of subunit vaccines. The Vi-capsular polysaccharide, one of the virulence factors is being exploited most in the development of subunit^10^, conjugates^11,12^ and multivalent vaccines^13^. Typherix^®^ (Vi-capsular polysaccharide vaccine), Peda-typhTM (conjugate vaccine) and Ty21a are commercially available parenteral vaccines that are also associated with some limitations such as local reactogenicity and multiple-dose regime^13^. Despite of tremendous efforts, the search for an effective vaccine against *Salmonella* is going on because of the above stated drawbacks of the existing vaccines^8,13,14^.

Typhoid toxin, decade ago identified as a potential virulence factor of *S*. Typhi and reported to play a role in the pathogenicity of *S*. Typhi^15^ can prove to be an effective vaccine candidate. In a persistent infection murine model, it has been observed that typhoid toxin is involved in the establishment of *S*. Typhi persistent infection most likely by altering the immune cell functions to its favor, although the complete underlying mechanism(s) is still not well understood^16,17^. Typhoid toxin has been documented to be produced intra-cellularly in the infected mammalian cells, which is then exported to the extracellular environment by a unique transport mechanism facilitated by *Salmonella*-containing vacuole^15,18^ (SCV). This mechanism helps toxin to traffic out of the infected cell to intoxicate other target cells, expressing the particular type of sialic acid which is abundantly expressed in humans. Typhoid toxin belongs to AB type toxin family with a slight difference as it has two components in A subunit instead of one, that is CdtB (cytolethal distending toxin B) and PltA (pertussis-like toxin A) and PltB (pertussis-like toxin B) as B subunits. CdtB and PltA form the active catalytic part of toxin while PltB helps in binding to the target cell^19^. CdtB subunit has a deoxyribonuclease like activity that inflicts DNA-damage thus inducing cell cycle arrest (mainly at G2 phase) and cellular distention thereby leading to apoptosis or senesence of the infected cell^18,20–22^. The potential link of typhoid toxin in its nano-bound form to the life-threatening symptomatology of typhoid fever suggests that it could serve as the basis for the development of sorely needed preventive strategy against the typhoid fever.

To the best of our knowledge, this is the first report indicating the immunoprotective potential of the CdtB subunit of the complexed typhoid toxin using a mouse peritonitis model with a view of intervening *Salmonella* infection.

## Material and Methods

### Bacterial strains

*Salmonella enterica* serovar Typhi strain Ty2 (initially procured from the CRI, Kasauli, India) and the clinical isolates (obtained from Government Medical College and Hospital, Sector- 32, Chandigarh and All India Institute of Medical Science, New Delhi) were used during the study. BL21 (DE3) *E.coli* competent host cell was used during the study. The bacterial cell suspension was prepared by culturing the cells overnight in Luria broth (pH 7.4) at 37 °C under constant shaking conditions (150 rpm) throughout the study^23,24^.

### Agents

Antibiotic kanamycin stock solution (50 mg/ml), Luria- Broth (LB) and agar from Himedia were used during the study. Enzymes such as Taq polymerase and restriction enzymes HindIII and NdeI were procured from Thermo Fishers. Isopropyl β-D-1-thiogalactopyranoside (IPTG) purchased from Sigma. Freund’s adjuvant procured for Sigma. Mouse cytokines kits: TNF-α was purchased from Krishgen Biosystems (Mumbai, India) and IL-6 and 10 from Diaclone, Besancon, France. Secondary anti-human and anti-mouse antibodies were purchased from Genei, Banglore, India.

### Cell-culture

Caco-2 and RAW 264.7 cell lines (obtained from National Centre for Cell Science (NCCS), Pune, India) were grown at 37 °C in humidified incubator with 5% CO_2_. The culture medium, Dulbecco’s Modified Eagle Medium (DMEM) supplemented with 5% fetal bovine serum, 50 U/ml of streptomycin and 100 U/ml of penicillin was changed every 2 days.

### Animals and ethical clearance

Animal studies were approved by the Animal Ethics Committee of Panjab University (approval no. PU/45/99/CPCSE/IAEC/2018/226). Inbred Balb/c female mice (6–8 weeks old) were used in the study. All animals were housed in clean polypropylene cages and fed standard antibiotic-free diet and water. All the experimental protocols were approved by Institutional Animal Ethics Committee (IAEC), Panjab University, Chandigarh (India) and performed in accordance with the guidelines of the Committee for the Purpose of Control and Supervision of Experiments on Animals (CPCSEA), Government of India. Animals were handled and disposed according to the guidelines of the Animal Institutional Ethical Committee, Panjab University, Chandigarh (India).

### *In-silico* studies and validating *CdtB* gene presence in *Salmonella* clinical isolates

In order to check the homology of the *CdtB* gene in various strains of *Salmonella* Typhi, gene sequence was subjected to BLASTn analysis (https://blast.ncbi.nlm.nih.gov/Blast.cgi). T cell and B cell immune epitopes of CdtB protein and T cell epitope-immunogenicity predictions were done using IEBD analysis resource (http://tools.iedb.org/main/)^25^. Further, the presence of *CdtB* gene was validated in *S*. Typhi Ty2 strain and 15 clinical isolates. To confirm the presence of this gene, PCR was run using forward 5′ TGGCATCGGACTGGGTAATG 3′ and reverse 5′ GTCTGTCTGGGCTGCGATTA 3′ primers at following conditions; Denaturation 95 °C for 30 seconds, annealing at 58 °C for 45 seconds and extension at 72 °C for 30 seconds.

### Plasmid construction and transformation

Pet28a, which is a widely used expression vector for the production of recombinant protein, was used in the present study. Briefly, *CdtB* gene was amplified using forward 5′ TAAGCACATATGATGAAAAAACCTGTTTTTTT 3′ and reverse primer 5′ TGCTTAAAGCTTTTAACAGCTTCGTGCCAAAA3′ having sites for HindIII and NdeI restriction enzymes, respectively. Amplified *CdtB* gene and vector were digested using thermo fast digest HindIII and NdeI enzymes. After digestion, both vector and gene were gel purified and ligation was done using Takara T4 DNA ligase enzyme at 16 °C for 30 min. Transformation of recombinant DNA was done into *E.coli* BL21(DE3) host cells using CaCl_2_ method^26^. Transformed cells obtained on kanamycin-containing Luria-agar plates were checked for the presence of recombinant plasmid by extracting plasmid by alkaline lysis method. To confirm the integrity of the inserted DNA into the pET28a plasmid nucleotide sequencing analysis was also done. The vector was extracted from transformed BL21 (DE3) *E.coli* cells, purified using Qiagen gel purification kit and was sequenced using T7 promoter and terminator primers.

### Expression and purification of the protein

For expression of CdtB protein, a single colony of transformed cells was inoculated in fresh antibiotic containing LB and incubated overnight at 37 °C under shaking condition. Next day 1% culture was inoculated in fresh antibiotic containing LB and allowed to grow at standard condition until O.D_600_ reaches to 0.4–0.6. 1 mM concentration of IPTG was used to induce protein expression.

His-tagged CdtB was purified from inclusion bodies by nickel-nitrilotriacetic acid (NTA)-agarose chromatography. Briefly, bacteria were harvested by centrifugation at 8000 rpm for 10 min, washed and suspended in binding buffer (20 mM Tris-Cl (pH 8), 5 mM imidazole, 500 mM NaCl). The suspension was sonicated for half an hour on ice and further centrifuged at 12000 rpm for 20 min to collect the inclusion bodies. The inclusion bodies were washed twice and suspended in 6 M urea. After incubation for an hour to dissolve the protein completely in urea, the suspension was centrifuged at 12000 g for 20 min. The obtained extract was applied to Ni-NTA column pre-equilibrated with binding buffer containing 6 M urea. The column was washed with the wash buffer (20 mM Tris-Cl (pH 8), 500 mM NaCl, 15 mM imidazole and 6 M urea). Bound protein was eluted with buffer containing 300 mM imidazole and further dialyzed against 10 mM Tris-Cl (pH 8) and 100 mM NaCl. The protein was concentrated using 10 kDa cut-off concentrators. The purity of the purified protein was checked using SDS-PAGE^27^.

### *In-vitro* nuclease activity of purified CdtB protein

To assess the *in-vitro* nuclease activity of CdtB protein, a protocol of Pons *et al*.^28^ was followed with few modifications. 200 ng of purified human DNA (extracted using Qiagen DNA extraction kit) was incubated at 37 °C with three different concentration of CdtB protein (100 ng, 250 ng and 500 ng) for 40 min in digestion buffer (50 mM NaCl, 20 mM Tris-HCl (pH 7.5), 5 mM CaCl2, 5 mM MgCl2, 50 μg/mL BSA). The reaction was stopped by adding 10 mM of EDTA and digestion products were analyzed on 1% agarose gel. Another experiment set with above-mentioned concentrations of protein was also performed in which incubation time was exceeded to 4 h and products were analyzed on 2% agarose gel, in order to observe digested DNA fragments (if any). DNA incubated at 37 °C in absence of CdtB and presences of bovine DNase I (2 ng for 10 min) were taken as negative and positive controls, respectively. The densitometry of DNA bands to determine % band intensity was done using ImageJ software.

### Evaluation of undesired effects of the toxin (if any) using various cell lines

#### Morphological alteration

To study the morphological alterations (if any) caused by CdtB protein in the eukaryotic cell, Caco-2 (human) and RAW 264.7 (mouse) cell lines were used. Briefly, 24 h semi-confluent cultures containing 4 × 10^4^ cells/ml were examined after 1 day of incubation in the presence of the protein preparation of different concentrations (1, 10 and 100 μg/ml). The cells were examined microscopically for any morphological alteration caused by CdtB protein at different concentrations^29^.

#### MTT (3-(4, 5-dimethylthiazol-2-yl)-2, 5-diphenyl tetrazolium bromide) assay

To measure the metabolic activity of cells (Caco-2 and Raw 264.7) in the presence of protein, MTT assay was done. Cells were harvested by trypsinization or scraping and the dilutions of the cells were made in culture medium up to 1 × 10^4^ cells/ml and plated out in triplicate into wells of a microtiter plate. The plate was then supplemented with different concentrations of the CdtB protein (1, 10 and 100 μg/ml) and incubated under appropriate conditions for 24 h. 100 μl of MTT (3-(4, 5-dimethylthiazol-2-yl)-2, 5-diphenyl tetrazolium bromide) solution was added and the plate was returned to cell culture incubator for 4 h. After 4 h, MTT solubilization buffer (100 μl) was added to each well and absorbance was recorded including the blank at 570 nm in a microtiter plate reader^30^.

#### Cell cycle analysis

Flow cytometry was done in order to investigate the effect of the protein on the cell cycle. Cells (Raw 264.7 and Caco-2) were seeded in 24-well plates at a density of 4 × 10^4^ cells/ml and treated with different concentrations of protein (1, 10 and 100 μg/ml) for 24 h. Cells were centrifuged, washed twice with PBS, suspended in 500 μl cold ethanol (70%) and incubated on ice for 15 min. After fixation, cells were suspended in 300 μl propidium iodide (PI) solution (0.05 mg/ml PI; 0.02 mg/ml RNase; 0.3% Triton X; 1 mg/ml sodium citrate) and re-incubated for 1 h on ice. Cells were washed with PBS and analyzed^29,31^.

### Evaluation of *in-vivo* immunogenicity of CdtB

#### Using sera of experimentally immunized mice

Potential of CdtB to act as an immunogen was checked in mice by injecting purified protein with an adjuvant. Primary dose (50 µg) of CdtB protein mixed with Freund’s complete adjuvant in 1:1 ratio was administered through intraperitoneal route to a group of 8 mice, followed by three booster doses of half the protein concentration (25 µg) mixed in 1:1 ratio with Freund’s incomplete adjuvant given at 14^th^, 21^th^ and 28^th^ day. In the control group, comprised of 4 mice, adjuvant mixed with PBS was injected. Sera collected from both, control and immunized group at day 17^th^, 23^th^, 30^th^ were subjected to quantitative (indirect ELISA) as well as qualitative (by western blotting, only after the second booster) analysis to measure and detect anti-CdtB IgG antibodies, respectively.

Briefly, 10 μg/ml of CdtB protein, diluted in carbonate buffer pH 9.6 was coated in the wells of microtitre plates overnight at 4 °C. Plates were washed thrice after every step with PBS pH 7.2 and blocked with 5% skim milk overnight. Next day, 100 μl serum samples at different dilution (made in blocking buffer) starting from 1: 500 to 1: 256000 were added to each well and incubated for 1 h at 37 °C. After that, 100 μl of anti-human IgG- HRP conjugated secondary antibody (1: 20000) was added to each well and incubated for 1 h at the same conditions. On completion of the incubation period, a substrate (TMB) 50 μl was added and allowed to develop color for 10 min in dark. The reaction was stopped with 50 μl/well of 0.1 N H_2_SO_4_, and the plate was read at 450 nm with a microplate reader^32,33^.

#### Western blotting

To detect CdtB specific antibody in the immunized mice, CdtB protein was run on 12% Tris-glycine SDS-PAGE gel and protein bands were transferred onto PVDF membrane. The membrane was blocked with 5% skimmed milk made in PBS-Tween 20 (0.05%) for 2 h at 37 °C. Two sera samples (obtained after the second booster) at 1: 500 dilutions (in blocking buffer) were incubated with the membrane for 1 h at 37 °C. The membrane was washed every time with wash buffer (PBS-T, 0.05%) and then incubated with anti-human IgG-HRP conjugated antibody (1: 20000 dilution) for 1 h at 37 °C. After this, the membrane was developed with chromogenic TMB solution (in the dark) for 10–15 min and the reaction was stopped by washing with wash buffer^32^.

### Establishment of *S*.Typhi Peritonitis mouse model

To establish the peritonitis model of *S*.Typhi (Ty2), the organism in 5% hog mucin was injected through intraperitoneal route^33–35^ at different doses (10^7^, 10^8^, 10^9^ CFU) in mice (n = 3) to standardize the dose that causes death within 24 h in order to understand the effect of vaccination on the survivability of the animals. Control group (n = 3) was administered with PBS.

### Active immunization

After checking the immunogenic potential of the protein, active immunization of two mice groups, comprised of 8 mice each was done. Immunization of mice was done in the same manner (at 1^st^, 14^th^, 21^st^ and 28^th^ day) as described in above section followed by challenge with a lethal dose (10^9^ CFU) of *S*.Typhi via intra-peritoneal route. With one group, the survival of the animals was recorded for the next 30 days post challenge and another group used for bacterial load studies, histological examination and cytokines level estimation. In the control group, comprised of 4 mice, only adjuvant mixed with PBS was administered at above stated days followed by the bacterial challenge (served as unimmunized-infected control).

#### Determination of bacterial load

Bacterial burden in the spleen and liver of control (unimmunized-infected) group at 20 h and in the immunized-infected group at 20 h and 7^th^ day post infection was checked. Mice were sacrificed by cervical dislocation and dissected under an aseptic condition to remove organs. Organs were suspended in sterile PBS (pH 7.2), homogenized, spread plated on MacConkey agar plates and kept at 37 °C for overnight incubation. The number of colony forming units was counted and expressed as log_10_ CFU.

#### Histoarchitectural studies

Liver and spleen specimens of unimmunized-uninfected, unimmunized-infected and immunized-infected groups removed aseptically (20 h and 7^th^ day in case of immunized-infected post infection) were fixed in 10% buffered formalin, stained with hematoxylin-eosin and observed under the microscope for assessing cellular infiltration and aggregation^36^.

#### Estimation of cytokines

Pro-inflammatory (TNF-α and IL-6) and anti-inflammatory (IL-10) cytokines in serum and organs (liver and spleen) were measured in control (unimmunized-infected) and immunized-infected groups at 20 h and 7^th^ day in case of immunized-infected post-infection using kits according to manufacturer’s instruction (TNF-α, Krishgen Biosystems, Mumbai, India^37^), IL-6 and 10, Diaclone, Besancon, Cedex, France^38,39^). Serum and organs were collected under aseptic conditions and processed further to measure cytokines levels^33,40^.

### Statistical analysis

Experiments were conducted at least three times and data are expressed as mean ± SD. Statistical analysis was done using GraphPad Prism 8 software by evaluating significance of data using Student’s t-test and one way analysis of variance (ANOVA). Values less than 0.05 (*p* < 0.05) were considered statistically significant.

## Results and Discussion

*Salmonella* Typhi and another emerging human restricted serovar *S*. Paratyphi A poses huge health challenges on developing nations. In addition to this, reports of MDR strains of *S*. Typhi have elevated the possibility of the recurrence of untreatable typhoid fever^5,41^. Currently, Ty21a and Vi-capsular polysaccharide-based vaccines (alone and conjugate; coupled to tetanus toxoid (TT) and HepatyrixTM) are available commercially^8,13^, but have some major limitations such as fail to provide complete protection against *S*.Typhi infection as Vi-antigen is not universally expressed by all the strains of *S*.Typhi^42,43^, inability to elicit an immune response in children below 2 years of age and local reactogenicity^13,14^. Clinical reports mainly from Asian countries, where typhoid fever is still a matter of concern, indicated around 55% and 52% efficacy of Vi-TT (tetanus toxoid) conjugate and Vi (polysaccharide) vaccines, respectively^44,45^; which has necessitated the need to look for other potent vaccine candidates against typhoid fever. A recent study has reported various new *S*.Typhi specific antigens^46^, one such novel antigen identified is CdtB, which constitutes an active motif of typhoid toxin and induces acute typhoid symptoms^47^. CdtB was selected for this study because the protein is unique as it is *S*.Typhi specific and is secreted post infection^48^.

**Figure 1:**
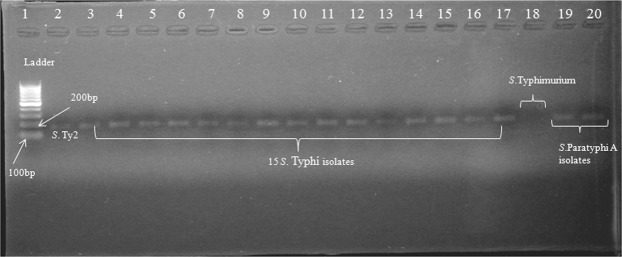
*CdtB* gene presence was confirmed in standard strain Ty2, 15 clinical isolates of *S*. Typhi and two *S*. Paratyphi isolates. Lane 1: 100 bp ladder (100–1000 bp), 2: *S*.Typhi Ty2, 3–17: 15 clinical isolates of *S*. Typhi, 18: *S*. Typhimurium and 19–20: *S*. Paratyphi A isolates (Full-length gel is presented in Supplementary Fig. S1).

To provide a broad range of protection to the host against the pathogen, the foremost requirement of any vaccine candidate is that it should be prevalent and conserved throughout the strains of specific species. BLASTn analysis indicated conservation of *CdtB* gene with 100% and 99% sequence homology in all strains of *S*.Typhi and *S*. Paratyphi A, respectively. Further, *CdtB* shares no similarity with human as well as mouse genome, which is crucial to avoid autoimmunity in the host. Various B and T cell (MHC class 1 and II) binding epitopes (peptides) of CdtB protein were predicted against different human HLA alleles in order predict immunogenic nature of protein; additionally, these predicted peptides could be exploited in the generation of epitope-based vaccines against typhoidal *Salmonella* (Suppl. Tables S1–S4). However, as it is not necessary that every epitope binding to MHC will be recognized by T-cells and generates an effective immune response, hence prediction of peptide-MHC (class I MHC) complexes to elicit a good immune response was also done, which showed good score against various CdtB peptides, indicating a greater probability of peptide-MHC complexes to elicit an immune response (Suppl. Table S1). Validation of the *in-silico* prediction of presence of *CdtB* gene done using 15 clinical isolates (of *S*.Typhi) showed gene prevalence in all along with two isolates of *S*.Paratyphi A (Fig. 1). However, it was found to be absent in *Salmonella* Typhimurium (Fig. 1), thereby supporting the fact of human restricted nature of “typhoidal Salmonella” as typhoid toxin is encoded by both typhoidal serovars *S*. Typhi and *S*. Paratyphi while it is mostly lacked by non-typhoidal serovars^49,50^.

**Figure 2:**
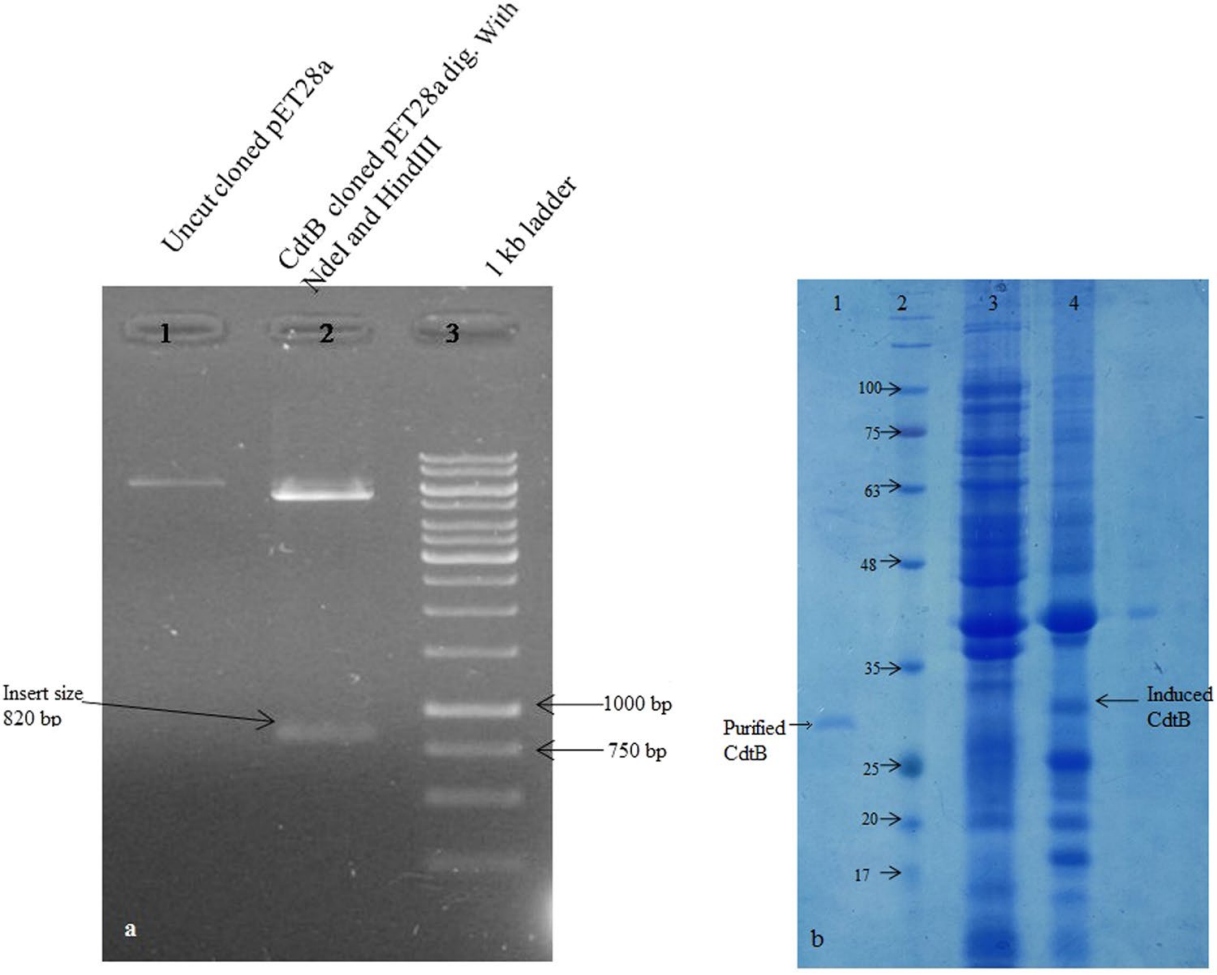
(**a**) Recombinant plasmid construction. Lane 1: Recombinant uncut plasmid, 2: Double digested (HindIII and NdeI) product of recombinant plasmid, 3: ladder (Full-length gel is presented in Supplementary Fig. S2a). (**b**) Showing the un-induced, induced BL21 cells at 37 °C and purified protein. Lane 1: Purified protein, 2: ladder (in kDa), 3: Uninduced cells and 4: Induced cells. (Uncropped, full-length gels are presented in Supplementary Fig. S2b).

As CdtB protein has been reported to be expressed under *in-vivo* condition only (inside the infected cells post-infection)^47^, therefore, in order to have protein in abundance for further studies, its gene was cloned into pET28a vector. Construction of recombinant plasmid was confirmed by restriction mapping of the positive cloned plasmid with NdeI and HindIII enzymes, as shown in Fig. 2a. Furthermore, nucleotide analysis of positive clones indicated 100% sequence homology with the *CdtB* gene of *S*.Typhi (Accession no. NC_003198.1), which signified the successful insertion of *CdtB* gene into the vector (Suppl. Fig. S3). Optimization of the protein expression was carried at different temperatures (28 °C, 37 °C and 40 °C) to maximize its production using 1 mM IPTG (Fig. 2b). As the protein was expressed in the insoluble fraction (as inclusion bodies), therefore it was purified under denaturing conditions^51^ using Ni-NTA affinity chromatography. After purification refolding of denatured protein to soluble form was done and final post-refolding yield of protein obtained was about 10 mg/liter culture. The purity of the purified protein was determined by Coomassie-stained SDS-PAGE gel analysis, which showed a band of approximately 31 kDa (Fig. 2b).

**Figure 3:**
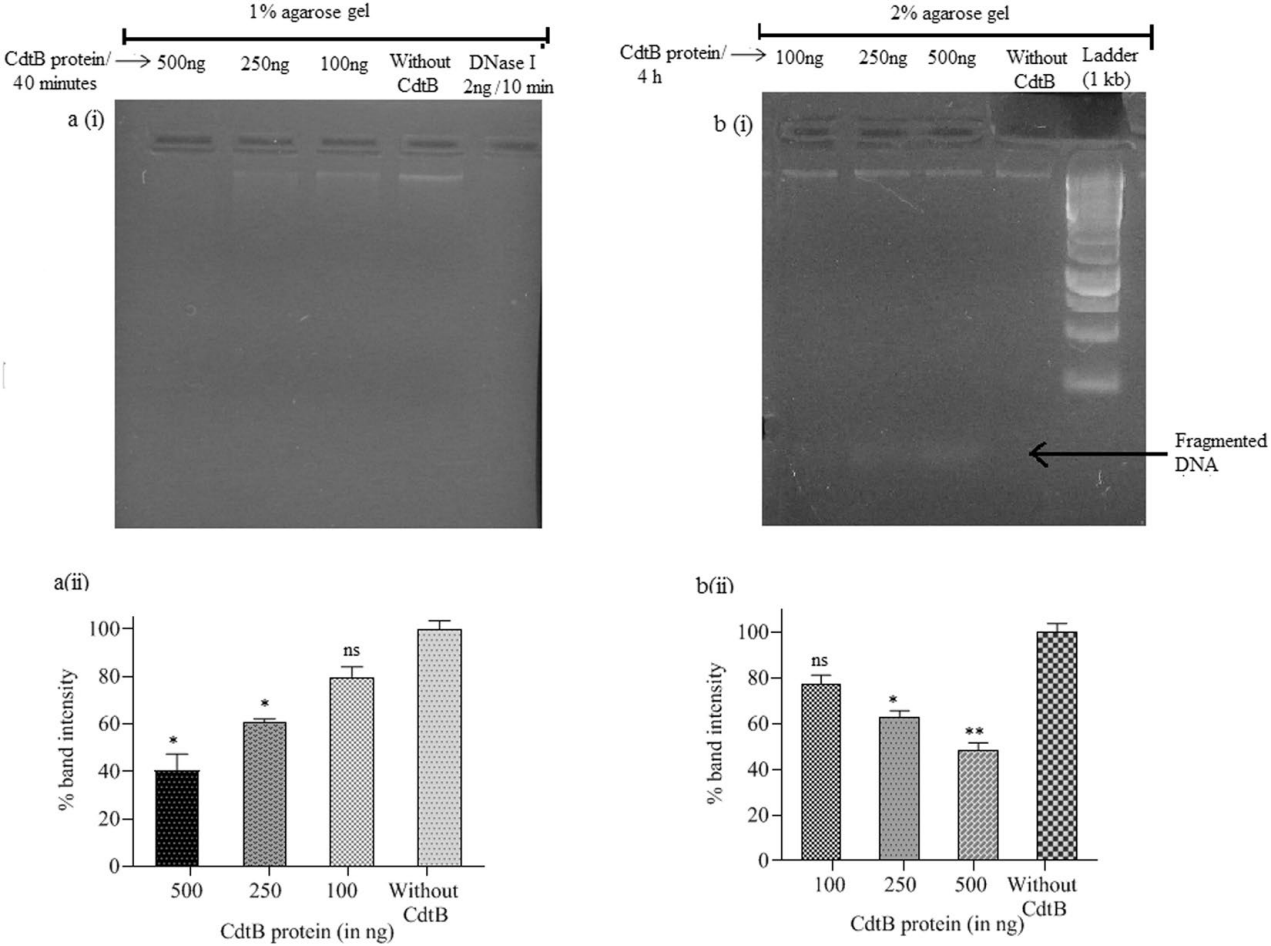
**a(i)** DNA run on 1% agarose gel after 40 min of incubation with different concentrations of CdtB protein and bovine DNase I (for 10 min) as positive control, **a(ii)** graph showing significant decrease in the % band intensity with increase in the concentration of protein, whereas DNase I (positive control) completely degraded the DNA within 10 min, **b(i)** DNA run on 2% agarose gel after 4 h of incubation with different concentrations of CdtB protein showing fragmented DNA and **b(ii)** graph showing significant decrease in the % band intensity with increase in the concentration of protein. The data are presented as mean ± SD. *p* value determined using one way ANOVA and t-test. *p < 0.05 and ns as non-significant as compared to control (without CdtB). (Full-length gels are presented in Supplementary Fig. S4).

**Figure 4:**
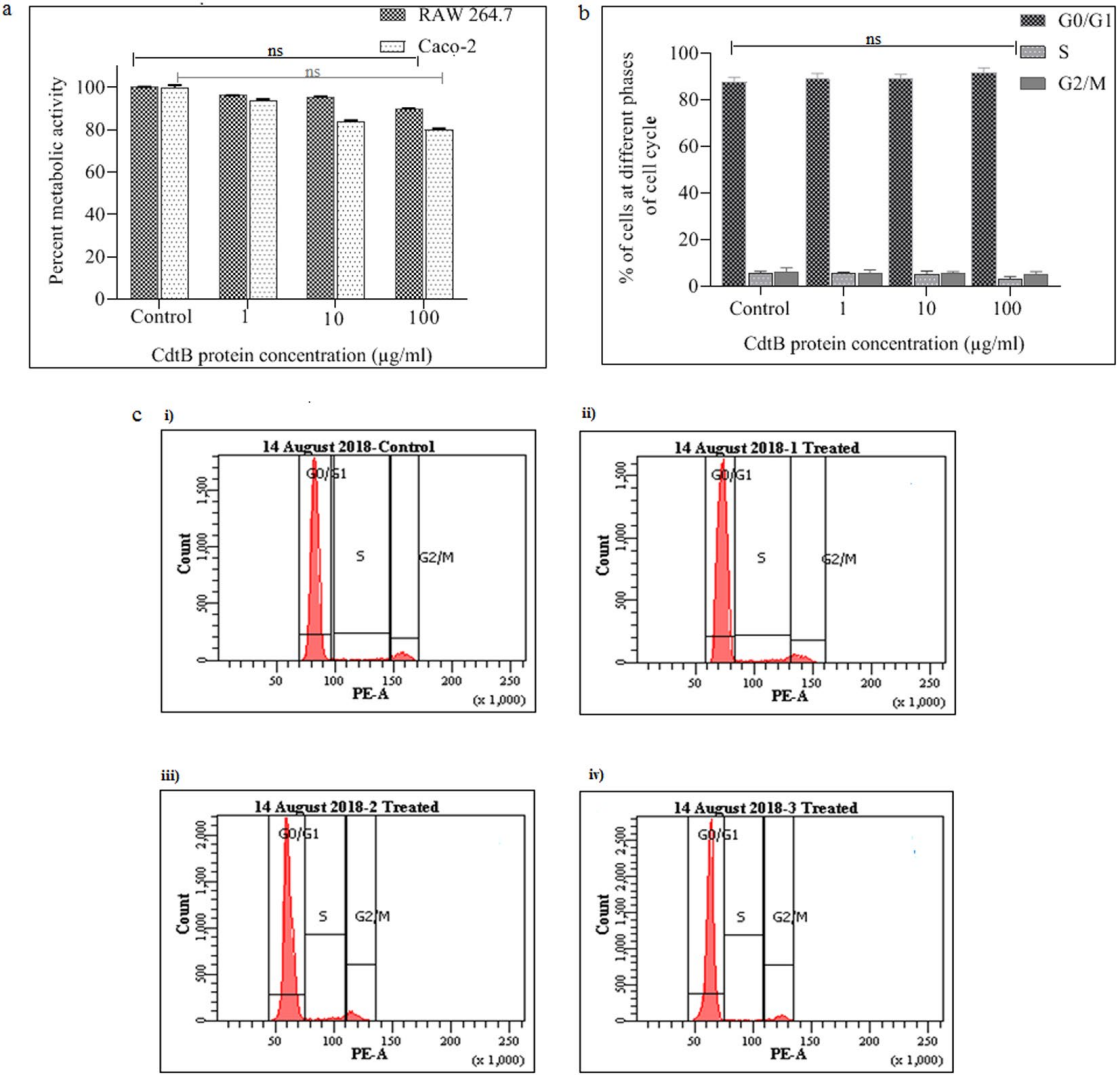
(**a**) Results of cell viability assay conducted on RAW 264.7 and Caco-2 cell lines. No significant metabolic inhibition was recorded in both cells. The data are presented as mean ± SD. *p* value determined using one-way ANOVA and t-test, ns as non-significant as compared to control. (**b**) Cell cycle analysis of RAW 264.7 cell lines. Purified protein at all the different concentrations (1, 10, and 100 µg/ml) showed no significant toxicity as the major population of the treated cells was found at Go/G1 phase of the cell cycle, similar to the one observed for control cells. The data are presented as mean ± SD. ns as non-significant as compared to control. (**c**) Representative figure of cell cycle analysis of RAW 264.7 cells, c (i) - control, (ii) 1 µg/ml, (iii) 10 µg/ml, (iv) 100 µg/ml.

CdtB shares position-specific sequence homology to the members of DNase I protein family^52^, which supports its nuclease activity^53,54^. *In-vitro* assessment of DNase activity of CdtB protein showed a significant decrease in the band intensity of DNA on 1% agarose gel (Fig. 3a(i,ii)), however no fragments of digested DNA was observed. In case of positive control complete degradation of DNA occurred within 10 min. In order to observe fragments of digested DNA, if any, products were analyzed on 2% gel after exceeding incubation time to 4 h. In the later set of experiment, fragmented DNA was observed with decrease in intensity of DNA (Fig. 3b(i,ii)), thereby indicating protein’s in*-vitro* nuclease activity. Under *in-vivo* conditions, CdtB subunit executes its DNase like catalytic activity (*in-vivo)* only along with other subunits PltA and PltB, which facilitate its entry inside the cell^29,49,55,56^. However, there are other reports, which describe the intoxication of cells with recombinantly produced CdtB protein of other Gram negative pathogens^57^. Therefore, before evaluating its immunoprotective efficacy in experimental animals, cytotoxicity studies of the subunit was still carried out in order to rule out its undesired effect. The sensitivity studies of different cultured cells such as RAW 264.7(mouse) and Caco-2 (human) to CdtB protein were conducted at different protein concentrations (1, 10 and 100 μg/ml). No significant changes were observed in the morphology of all cell types (Suppl. Fig. S5) and no inhibition of metabolic activity were found in mouse cell lines compared to human cell line, where slight concentration-dependent inhibition of metabolic activity was recorded (Fig. 4a). Encouraged by these findings and keeping in view the relevance of animal model adopted for the study, cell cycle analysis was performed with RAW 264.7 cells. The results revealed that purified protein at all the used concentrations showed insignificant toxicity, as major population of cells was found at G_o_/G_1_ phase of the cell cycle same like control cells (Fig. 4b,c). Similar observations were obtained with Caco-2 cell lines and the results are provided in supplementary information (Suppl. S6). Thus the flow cytometric analysis further strengthened our former observations and was also found in agreement with the earlier findings, wherein it has been reported that all subunits of the toxin are required for intoxication of cells^29,31,49^.

**Figure 5:**
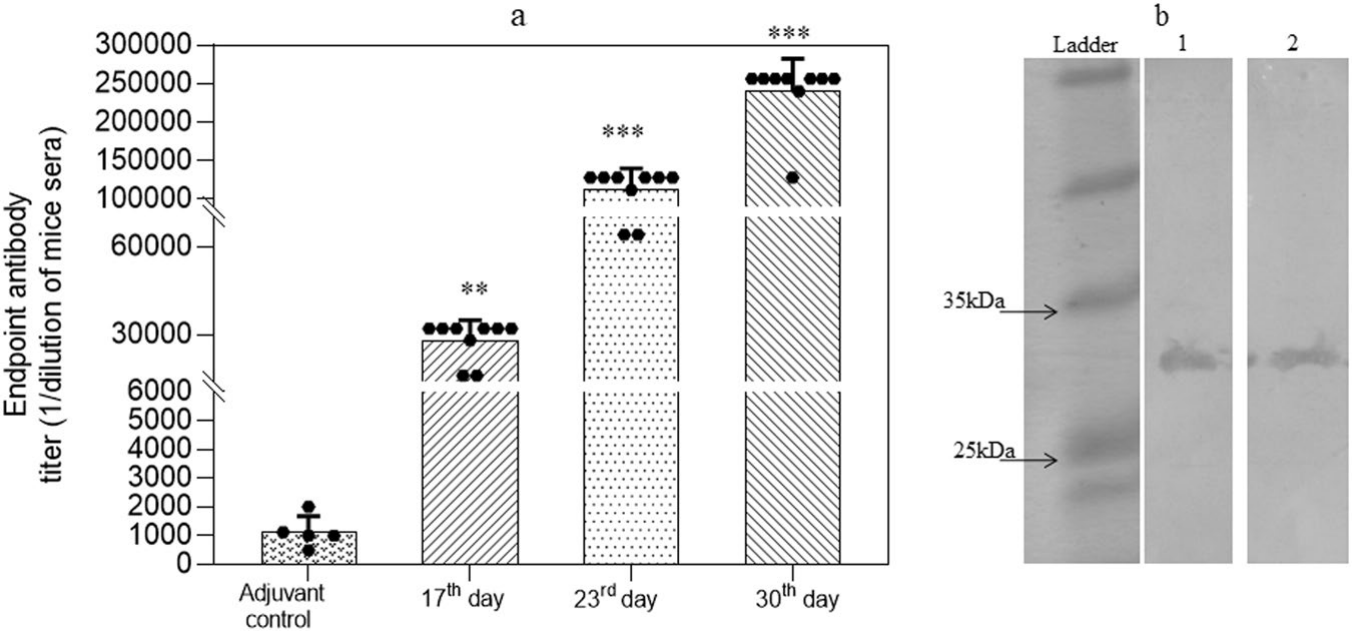
(**a**) Endpoint antibody titer measured after every booster at 17^th^, 23^th^ and 30^th^ day (n = 8). Black dots represent each mouse and bars represent mean value of all mice plus SD. The data are presented as mean ± SD. *p* value determined using one way ANOVA and t-test, *p < 0.05, **p < 0.01 and ***p < 0.001 as compared to adjuvant control. (**b**) Western blot of two serum samples of mice (sera dilution used 1:500) after the second booster showed evident blot, indicating the generation of anti-CdtB antibodies (Full-length image is presented in Supplementary Fig. S7).

In reference to the humoral response, the significant titer of antibodies to typhoid toxin has been reported in convalescent typhoid fever patients^46,58^. In order to detect the antibody titer particularly against purified CdtB protein (anti-CdtB antibodies), mice were administered (intra-peritoneal) with CdtB protein along with Freund’s adjuvant to achieve the sustained release of protein; as it is considered as most effective for generating an immune response to diverse antigens^59^. CdtB protein generated antibody titer of >25000 (±7406 standard deviation) after first booster and >100000 (±29626 standard deviation) and >225000 (±45254 standard deviation) after the second and third booster, respectively, whereas in the case of control (adjuvant only) no considerable titer was recorded. A significant titer of IgG antibodies evoked after immunization with CdtB indicated its fairly good immunogenic potential in mouse (Fig. 5a). Western blot analysis of two serum samples done after the second booster further confirmed the presence of anti-CdtB antibodies in the sera (Fig. 5b), thereby indicating the seroreactivity of CdtB; thus signifying its application in the development of a point-of-care. During and after completion of immunization no ill-effect of immunization was observed as mice remained healthy, behaviorally active and organs were found completely sterile.

After checking the *in-vivo* immunogenicity of CdtB protein, immunization studies were carried out. To monitor the immunoprotective efficacy of CdtB, peritonitis murine model was established. As *S*.Typhi causes self-limiting infection in mice with low bacterial load in organs and low dissemination, therefore, 5% hog mucin was used to increase the virulence of organisms as reported earlier^33,60,61^. Out of the three different doses given to mice, 10^9^ CFU dose led to 100% mortality of mice within 24 hours of infection. Therefore, this dose was selected and used to carry out the efficacy studies of the protein against this challenge dose.

**Figure 6:**
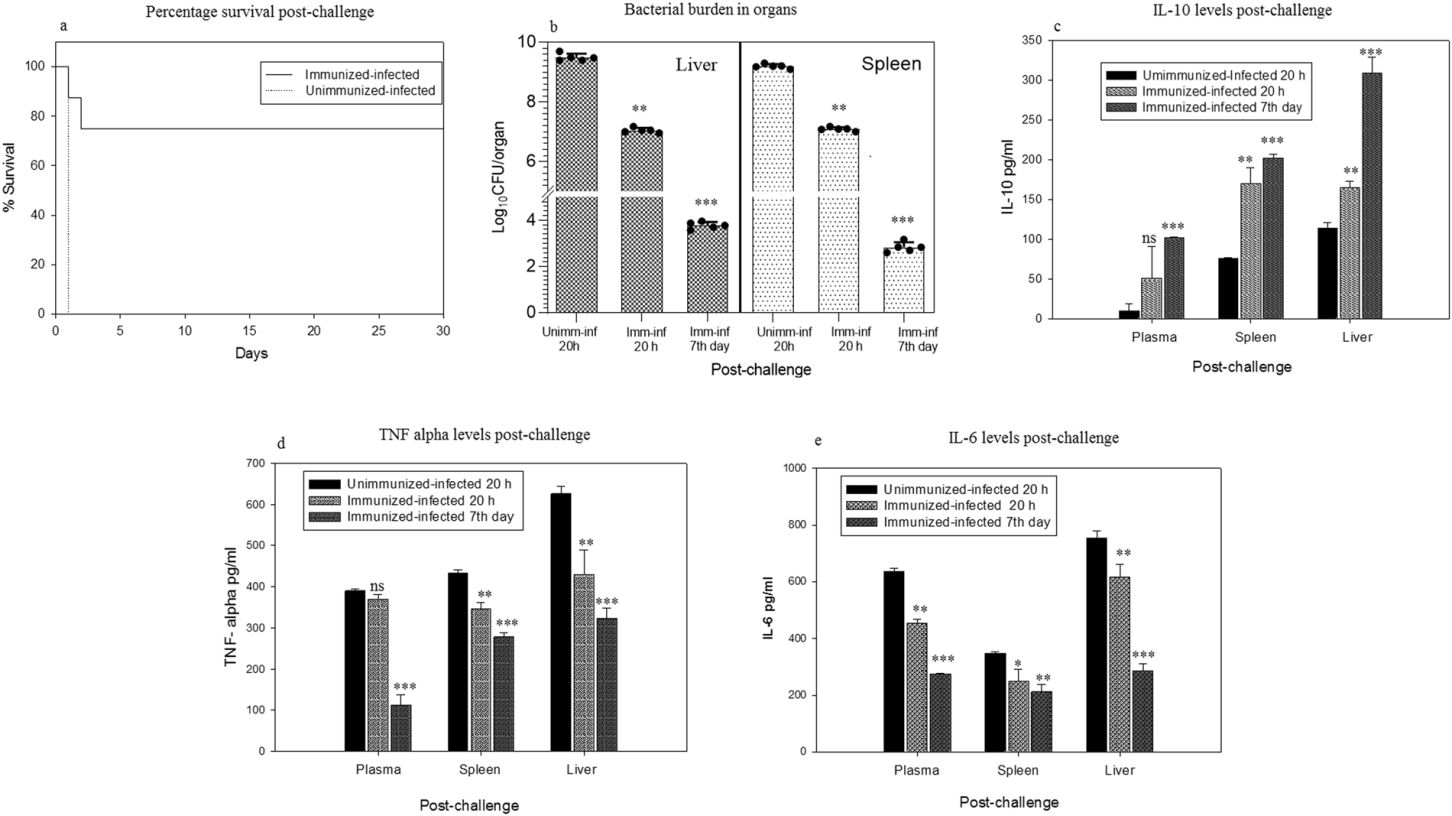
(**a**) Percentage survival of mice of the immunized-infected group post-challenge (n = 8), recorded up to 30 days. (**b**) Bacterial load detected in the spleen and liver of control (Unimm-Inf 20 h) and immunized-infected (Imm-Inf 20 h) mice at 20 h post-challenge and at 7^th^ day in immunized-infected (Imm-Inf 7^th^ day) showing significant log reduction. Black dots represent each mouse and bars represent mean value plus SD. (**c**) IL-10, anti-inflammatory cytokine measured in serum, spleen and liver of control (unimmunized-infected) and immunized-infected mice at 20 h post-infection and at 7^th^ day in immunized-infected mice. (**d,e**) TNF-alpha and IL-6 levels measured in serum, spleen and liver of the control (unimmunized-infected) and immunized-infected mice at 20 h post-infection and at 7^th^ day in immunized-infected mice, respectively. The data are presented as mean ± SD. *p* value determined using one way ANOVA and t-test, *p < 0.05, **p < 0.01 and ***p < 0.001 and ns as non-significant as compared to control (unimmunized-infected) mice.

Mice survival recorded up to thirty days post-challenge showed 75% survival in the immunized-infected group, whereas in the control group (unimmunized-infected) all mice died within 24 h (Fig. 6a). Active immunization of mice followed by bacterial challenge not only increased the mice survival rate but also led to the significant decrease in the bacterial burden as evident by bacterial load studies (Fig. 6b). At 20 h post challenge, bacterial load was checked in the liver and spleen. In the liver, approximately 9 log and 7 log CFU was recorded in control (unimmunized-infected) and immunized-infected mice, respectively, indicating a significant decrease of two log folds in immunized mice. Similarly, bacterial count checked at 7^th^ day in immunized-infected mice showed almost five log reduction. The same trend was observed in the case of spleen also, where a significant reduction in the bacterial burden was recorded in the immunized-infected group as compared to the control (unimmunized-infected, Fig. 6b).

**Figure 7:**
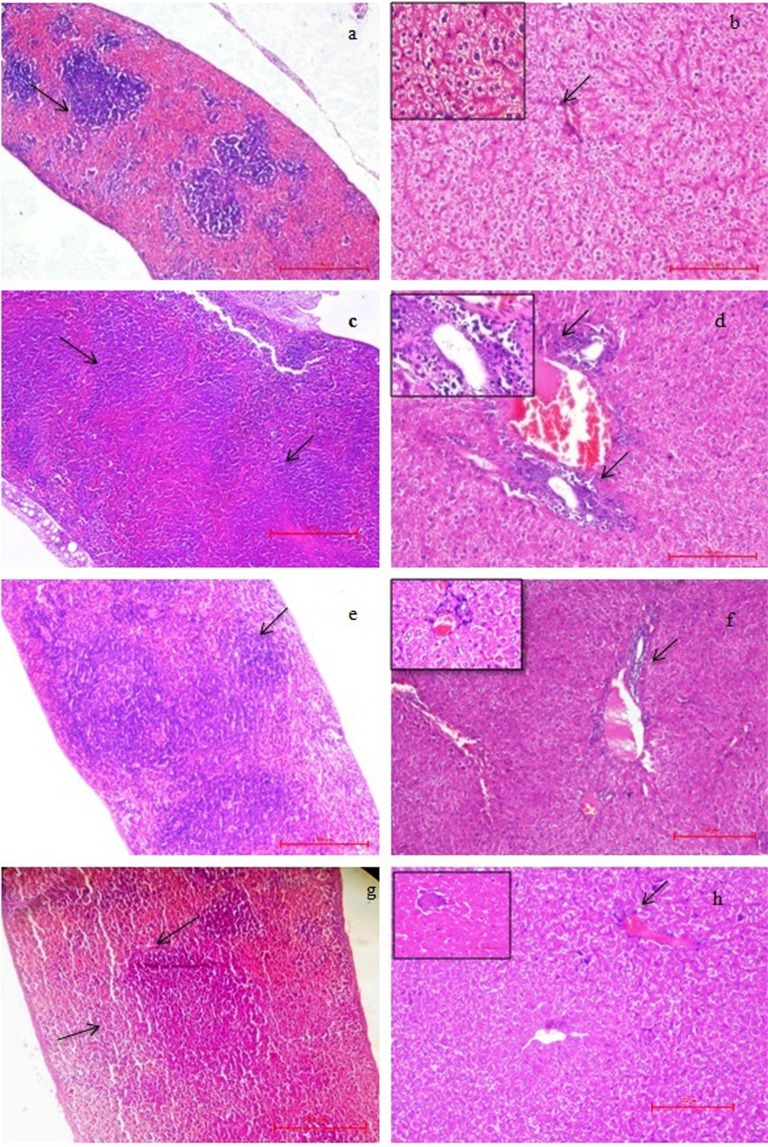
Histopathology of the spleen (40X) and liver (100X). (**a**) Spleen of unimmunized-uninfected mice showed a normal structure with clearly distinct red pulp, white pulp and marginal zone, score 0. (**b**) Liver of unimmunized-uninfected showed the normal size of hepatocytes and normal portal tract indicated with an arrow, score 0 (inset 400X). (**c**) Spleen of the control (unimmunized-infected) mice, 20 h post-challenge showed an increased number of lymphoid follicles with the disappearance of red pulp and enlargement of size, score 4. (**d**) Liver from control (unimmunized-infected, 20 h) showed lymphocytic infiltration near portal tracts, indicated with arrows, score 3 (inset 400X). (**e**) Immunized-infected (20 h post-challenge) spleen showed rehabilitation of red and white pulp, score 2. (**f**) Immunized-infected liver (20 h)) showed less number of inflammatory cells near portal tracts (indicated with an arrow) with restoration of histological structure, score 2 (inset 400X). (**g,h**) spleen (score 1) and liver (score 1) of immunized-infected mice at 7^th^ day showing restored histoarchitecture. (High quality image is provided in supplementary information, Fig. S8).

Tissue architecture studies and cytokines level estimation (pro and anti-inflammatory) serve as a good indicator to study the initiation of infection, inflammation and effectiveness of any treatment or vaccine^37,62^. Histology of spleen and liver of the control (unimmunized-infected) group showed signs of active infection 20 h post challenge. In the case of spleen, hyperplasia (increased in number) of lymphoid follicles and enlargement of size (hypertrophy) was observed with a reduction of red pulp (score 4, after giving challenge to unimmunized-infected mice, Fig. 7c) as compared to the normal spleen of unimmunized-uninfected mice where distinct follicles with clear red and white pulp could be seen, score 0 (Fig. 7a). However, the red and white pulp of the spleen of immunized-infected mice at 20 h (score 2) and 7^th^ day (score 1) post-infection appeared to be restored (Fig. 7e,g). In context to the liver, portal triaditis was seen in control (unimmunized-infected) group post challenge compared to unimmunized-uninfected mice (score 0, Fig. 7b), which indicated inflammation as both portal tracts showed lymphocytic infiltrations (score 3, Fig. 7d). However, the immunized-infected group showed reduced inflammation with a clearance of portal tracts after 20 h (score 2) post-infection (Fig. 7f). Liver of immunized-infected mice at 7^th^ day showed normal hepatic structure (score 1, Fig. 7h).

Cytokines play a key role in the development of disease and in providing immunity against the infection. Both the anti and pro-inflammatory cytokines are important in determining the protection mediated by the immune system against the specific pathogenic organisms^40,63^. Pro-inflammatory cytokines such as TNF-α, IL-6, Interferon-γ, etc., are signaling molecules secreted by the immune cells, which promote inflammation and play an important role in mediating the innate immune response^23,40^. TNF-α acts as a local inflammatory mediator and activates B and T cells to produced IL-6. On the other hand, anti-inflammatory cytokines (such as IL-10) are a series of immunoregulatory molecules that down-regulate inflammatory reactions^64^. Rise in the levels of anti-inflammatory cytokine IL-10 in sera as well as in tissue homogenates (liver and spleen) of immunized-infected group was detected post 20 h and 7^th^ day of post- challenge, indicating the employment of immune cells to fight against the pathogen^64^ that was in contrast to the control (unimmunized-infected) group (Fig. 6c). Increased levels of pro-inflammatory cytokines such as TNF-α and IL-6 in control (unimmunized-infected) group indicated the active infection, which could be linked with the infiltration of lymphocytes in the liver, increase in number of lymphoid follicles in the spleen and heavy bacterial load in the organs; whereas in immunized-infected group significant decrease in the levels of TNF-α and IL-6 (at 20 h and 7^th^ day post infection) signified the efficacy of CdtB as vaccine (Fig. 6d,e). These findings are in accordance with the earlier reports wherein cytokine-producing Th type cell-response has been documented to play a critical role in providing resistance to *S*.Typhi infection at the systemic level^41^. This study also indicates the involvement of Th cells to stimulate the B- cells for the production of antibodies as well as the cytokines influenced activation of macrophages residing in the reticuloendothelial system which are expected to play a critical role in clearing systemic *S*.Typhi infection. This study thus indicates that the protein is capable of eliciting the humoral as well as a cellular immune response against the infection.

It may be concluded from the study that successfully cloned and purified CdtB protein showed an immunoprotective response, as evident by the antibody titer and cellular immune response generated against the disease. Immunization with the protein conferred 75% protection against the lethal dose of *S*.Typhi with a reduction in bacterial burden, levels of pro-inflammatory cytokine and restoration of histoarchitecture. Thus, the proof of concept of this study may prove to be helpful in further developing it as a prophylactic option not only against *S*. Typhi but also against *S*. Paratyphi A, for which no vaccine is available commercially.

## Supplementary information

supplementary information

## Data Availability

All the relevant data are within the manuscript and in the supplementary information.
