# Author Correction: Prophylactic potential of cytolethal distending toxin B (CdtB) subunit of typhoid toxin against Typhoid fever

**DOI:** 10.1038/s41598-020-59362-z

**Published:** 2020-02-19

**Authors:** Reena Thakur, Preeti Pathania, Navneet Kaur, Vattan Joshi, Kanthi Kiran Kondepudi, Chander Raman Suri, Praveen Rishi

**Affiliations:** 1 Department of Microbiology, Panjab University, Chandigarh, India; 2 National Agri-Food Biotechnology Institute, Mohali, India; 3 Indian Institute of Technology Ropar, Rupnagar, Punjab India

Correction to: *Scientific Reports* 10.1038/s41598-019-54690-1, published online 05 December 2019

The original version of this Article contained a typographical error in the spelling of the author Chander Raman Suri, which was incorrectly given as Raman Chander Suri. This has now been corrected in the PDF and HTML versions of the Article, and in the accompanying Supplementary Information file.

Additionally, the original version of this Article contained a typographical error in the Author Contributions section.

“P.R. conceived the idea and organized the study. R.T. performed all the experiments and wrote the manuscript. K.K.K. provided cell culture facility. P.P., N.K. and V.J. helped in protein purification and animal work, respectively. P.R. and R.C.S. critically reviewed and approved the manuscript.”

now reads:

“P.R. conceived the idea and organized the study. R.T. performed all the experiments and wrote the manuscript. K.K.K. provided cell culture facility. P.P., N.K. and V.J. helped in protein purification and animal work, respectively. P.R. and C.R.S. critically reviewed and approved the manuscript.”

