# Supplementary material for: Prophylactic potential of cytolethal distending toxin B (CdtB) subunit of typhoid toxin against Typhoid fever
Source: Sci Rep. 2019 Dec 5;9:18404. doi: 10.1038/s41598-019-54690-1 (PMC6895121; doi:10.1038/s41598-019-54690-1)

Supplementary Information

Prophylactic potential of cytolethal distending toxin B (CdtB) subunit of typhoid toxin against Typhoid fever

Reena Thakur[1], Preeti Pathania[1], Navneet Kaur[1], Vattan Joshi[1], Kanthi Kiran Kondepudi[2], Raman Chander Suri[3*] and Praveen Rishi[1*]

[1]Department of Microbiology, Panjab University, Chandigarh, India
[2]National Agri-Food Biotechnology Institute, Mohali, India
[3]Indian Institute of Technology Ropar, Rupnagar, Punjab, India

Table-S1: MHC Class-I binding peptides (of CdtB) predicted using IEDB software. Peptides having percentile rank lower than 0.5 (a small numbered percentile rank indicates high binding affinity) are listed. As it is not necessary that every peptide epitope binding to MHC-I will induce good immune response, therefore T-cell epitope-immunogenicity of listed peptides were also checked (higher score indicates better ability to elicit immune response).

Alleles	Start	End	Peptides	Method	Percentile rank	T-cell epitope-immunogenicity score
HLA-B*15:01	10	19	TMIICSYISF	Consensus (ann/smm)	0.12	-0.024
HLA-B*07:02	17	26	RPTTVASRPV	Consensus (ann/smm)	0.14	**0.01143**
HLA-B*15:01	7	16	FLLTMIICSY	Consensus (ann/smm)	0.15	-0.02282
HLA-B*58:01	36	45	GSSASTESKW	Consensus (ann/smm)	0.17	-0.309
HLA-A*68:01	51	59	SAIDVGARR	Consensus (ann/smm)	0.18	**0.20088**
HLA-B*58:01	24	32	ISDYKVMTW	Consensus (ann/smm)	0.2	-0.29318
HLA-B*58:01	37	45	SSASTESKW	Consensus (ann/smm)	0.2	-0.28734
HLA-A*68:01	50	59	HSAIDVGARR	Consensus (ann/smm)	0.2	**0.30358**
HLA-A*01:01	39	47	TTSRQDIRY	Consensus (ann/smm)	0.21	**0.04902**
HLA-A*02:01	14	22	QLASDHYPV	Consensus (ann/smm)	0.22	-0.11132
HLA-B*57:01	37	45	SSASTESKW	Consensus (ann/smm)	0.22	-0.28734
HLA-A*33:01	10	18	WFLAGDFNR	Consensus	0.22	**0.18467**

				(ann/smm)		
HLA-A*68:01	19	28	FACANISDYK	Consensus (ann/smm)	0.23	**0.006**
HLA-A*02:03	27	35	LMTEHLERV	Consensus (ann/smm)	0.23	**0.24915**
HLA-A*33:01	18	27	DHYPVAFLAR	Consensus (ann/smm)	0.28	**0.18964**
HLA-B*15:01	11	19	MIICSYISF	Consensus (ann/smm)	0.3	-0.15997
HLA-A*02:03	14	22	QLASDHYPV	Consensus (ann/smm)	0.32	-0.11132
HLA-B*07:02	1	10	APYSQRVEAL	Consensus (ann/smm)	0.33	-0.08385
HLA-A*02:06	50	59	RQLLSGTAGV	Consensus (ann/smm)	0.34	-0.0535
HLA-A*24:02	15	24	SYISFACANI	Consensus (ann/smm)	0.35	**0.00732**
HLA-B*57:01	24	32	ISDYKVMTW	Consensus (ann/smm)	0.35	-0.29318
HLA-B*15:01	45	54	TQIGGGILDY	Consensus (ann/smm)	0.36	**0.27218**
HLA-A*31:01	9	17	RADNVYVLR	Consensus (ann/smm)	0.36	**0.06577**
HLA-A*02:06	27	35	LMTEHLERV	Consensus (ann/smm)	0.36	**0.24915**
HLA-A*33:01	33	42	YTWNLGTTSR	Consensus (ann/smm)	0.36	**0.06023**
HLA-B*58:01	26	35	VGIPIDEYTW	Consensus (ann/smm)	0.37	**0.29705**
HLA-A*33:01	34	42	TWNLGTTSR	Consensus (ann/smm)	0.39	-0.00762
HLA-B*51:01	9	17	LTMIICSYI	Consensus (ann/smm)	0.4	**0.01399**
HLA-B*51:01	22	30	QPFGVGIPI	Consensus (ann/smm)	0.4	**0.25004**
HLA-B*35:01	19	27	FACANISDY	Consensus (ann/smm)	0.4	**0.01419**
HLA-A*30:01	7	15	RQRADNVYV	Consensus (ann/smm)	0.4	**0.01419**
HLA-A*26:01	11	19	MIICSYISF	Consensus (ann/smm)	0.4	-0.15997
HLA-A*33:01	3	11	YSQRVEALR	Consensus (ann/smm)	0.41	**0.17547**
HLA-A*11:01	36	44	GSSASTESK	Consensus (ann/smm)	0.42	-0.15105
HLA-A*23:01	15	24	SYISFACANI	Consensus (ann/smm)	0.46	**0.00732**
HLA-B*53:01	28	37	IPIDEYTWNL	Consensus (ann/smm)	0.46	**0.37912**
HLA-B*35:01	24	33	FGVGIPIDEY	Consensus (ann/smm)	0.48	**0.3688**

HLA-A*02:03	36	45	FLTAHALASG	Consensus (ann/smm)	0.48	**0.04749**
HLA-A*01:01	16	24	ASDHYPVAF	Consensus (ann/smm)	0.48	**0.08341**
HLA-A*68:01	42	50	ESKWNVNVR	Consensus (ann/smm)	0.49	**0.20411**
HLA-A*68:02	9	17	LTMIICSYI	Consensus (ann/smm)	0.5	**0.01399**
HLA-B*51:01	39	47	LAPTEPTQI	Consensus (ann/smm)	0.5	**0.0876**

Table S2: T-cell epitope-immunogenicity of complete CdtB protein was also checked. Higher Score indicates greater probability of generating immune response.

**Peptide**	**length**	**score**
NRRADEVFVLSPVRQGGRPLLGIRIGNDAFFTAHAIAMRNNDAPALVEEVYNFFRDSRDP	60	1.62838
MKKYIISLIVFLSFYAQADLTDFRVATWNLQGASATTESKWNINVRQLISGENAVDILAV	60	1.01696
VHQALNWMILGDFNREPADLEMNLTVPVRRASEIISPAAATQTSQRTLDYAVAGNSVAFR	60	0.69675
QEAGSPPSTAVDTGTLIPSPGIPVRELIWNLSTNSRPQQVYIYFSAVDALGGRVNLALVS	60	0.49979
PSPLQAGIVYGARRTQISSDHFPVGVSRR	29	0.17302

Table S3: MHC Class- II binding peptides predicted against frequently occurring human HLA alleles using IEDB software, peptides having percentile rank lower than 0.5 are listed below.

Allele	Start	End	Peptide	Method	Percentile_ rank
HLA-DRB1*03:01	230	244	GILDYGVIVDRAPYS	Consensus (smm/nn/sturniolo)	0.08
HLA-DRB1*03:01	231	245	ILDYGVIVDRAPYSQ	Consensus (smm/nn/sturniolo)	0.08
HLA-DRB1*03:01	232	246	LDYGVIVDRAPYSQR	Consensus (smm/nn/sturniolo)	0.08
HLA-DRB1*03:01	233	247	DYGVIVDRAPYSQRV	Consensus (smm/nn/sturniolo)	0.08
HLA-DRB1*03:01	234	248	YGVIVDRAPYSQRVE	Consensus (smm/nn/sturniolo)	0.08
HLADPA1*03:01/ DPB1*04:02	1	15	MKKPVFFLLTMIICS	Consensus (smm/nn/sturniolo)	0.1
HLADPA1*03:01/ DPB1*04:02	2	16	KKPVFFLLTMIICSY	Consensus (smm/nn/sturniolo)	0.15
HLA-DRB1*03:01	235	249	GVIVDRAPYSQRVEA	Consensus (smm/nn/sturniolo)	0.17
HLADPA1*03:01/ DPB1*04:02	3	17	KPVFFLLTMIICSYI	Consensus (smm/nn/sturniolo)	0.18
HLA-DRB1*03:01	236	250	VIVDRAPYSQRVEAL	Consensus (smm/nn/sturniolo)	0.18
HLADQA1*04:01/ DQB1*04:02	163	177	ASGGPDAAAIVRVTI	Consensus (smm/nn/sturniolo)	0.21
HLADQA1*04:01/ DQB1*04:02	162	176	LASGGPDAAAIVRVT	Consensus (smm/nn/sturniolo)	0.22
HLA- DPA1*03:01/DPB 1*04:02	4	18	PVFFLLTMIICSYIS	Consensus (smm/nn/sturniolo)	0.28
HLA-DRB1*08:02	132	146	NVYVLRPTTVASRPV	Consensus (smm/nn/sturniolo)	0.32
HLA-DRB1*11:01	1	15	MKKPVFFLLTMIICS	Consensus (smm/nn/sturniolo)	0.34
HLA-DRB1*11:01	2	16	KKPVFFLLTMIICSY	Consensus (smm/nn/sturniolo)	0.34
HLA-DRB1*11:01	3	17	KPVFFLLTMIICSYI	Consensus (smm/nn/sturniolo)	0.34
HLA-DRB1*08:02	130	144	ADNVYVLRPTTVASR	Consensus (smm/nn/sturniolo)	0.34
HLA-DRB1*08:02	131	145	DNVYVLRPTTVASRP	Consensus (smm/nn/sturniolo)	0.36
HLADQA1*04:01/ DQB1*04:02	161	175	ALASGGPDAAAIVRV	Consensus (smm/nn/sturniolo)	0.37

HLA-DPA1*03:01/DPB1*04:02	5	19	VFFLLTMIICSYISF	Consensus (smm/nn/sturniolo)	0.4
HLA-DRB1*07:01	101	115	SRQDIRYIYHSAIDV	Consensus (smm/nn/sturniolo)	0.43
HLA-DRB1*11:01	4	18	PVFFLLTMIICSYIS	Consensus (smm/nn/sturniolo)	0.43
HLA-DRB1*08:02	133	147	VYVLRPTTVASRPVI	Consensus (smm/nn/sturniolo)	0.43
HLA-DRB1*08:02	209	223	TEHLERVVAVLAPTE	Consensus (smm/nn/sturniolo)	0.43
HLA-DRB1*12:01	3	17	KPVFFLLTMIICSYI	Consensus (smm/nn/sturniolo)	0.44
HLA-DRB1*07:01	102	116	RQDIRYIYHSAIDVG	Consensus (smm/nn/sturniolo)	0.46
HLA-DRB1*07:01	103	117	QDIRYIYHSAIDVGA	Consensus (smm/nn/sturniolo)	0.46
HLADQA1*03:01/DQB1*03:02	53	67	LSGTAGVDILMVQEA	Consensus (smm/nn/sturniolo)	0.48
HLA-DRB1*08:02	208	222	MTEHLERVVAVLAPT	Consensus (smm/nn/sturniolo)	0.48
HLA-DPA1*03:01/DPB1*04:02	203	217	LENDLMTEHLERVVA	Consensus (smm/nn/sturniolo)	0.49
HLA-DRB1*12:01	2	16	KKPVFFLLTMIICSY	Consensus (smm/nn/sturniolo)	0.5

Table S4: Linear B-cell epitopes predication (of CdtB protein) was done using (Bepipred Linear Epitope Prediction 2.0) IEDB analysis software. Scores above threshold score (0.50) are predicted as a part of an epitope and colored in yellow on the graph below.

Start	end	Peptide epitopes	length
5	10	VFFLLT	6
14	83	CSYISFACANISDYKVMTWNLQGSSASTESKWNVNVRQLLSGTAGVDILMV QEAGAVPTSAVPTGRHIQP	70
93	133	YTWNLGTTSRQDIRYIYHSAIDVGARRVNLAIVSRQRADNV	41
135	135	V	1
138	172	PTTVASRPVIGIGLGNDVFLTAHALASGGPDAAAI	35
185	215	MRHLSWFLAGDFNRSPDRLENDLMTEHLERV	31
222	235	TEPTQIGGGILDYG	14
241	258	APYSQRVEALRNPQLASD	18
261	261	P	1

Predicted epitopes colored in yellow

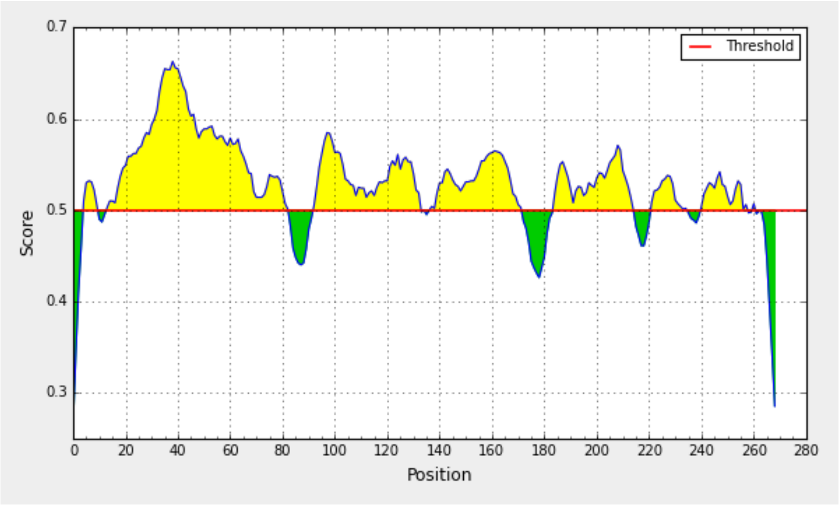

Figure S1. Original image *CdtB* gene presence was confirmed in standard strain Ty2, 15 clinical isolates of *S*. Typhi and two *S*. Paratyphi isolates. Lane 1: 100 bp ladder (100-1000 bp), 2: *S*.Typhi Ty2, 3-17: 15 clinical isolates of *S*. Typhi, 18: *S*. Typhimurium and 19 - 20: *S*. Paratyphi A isolates

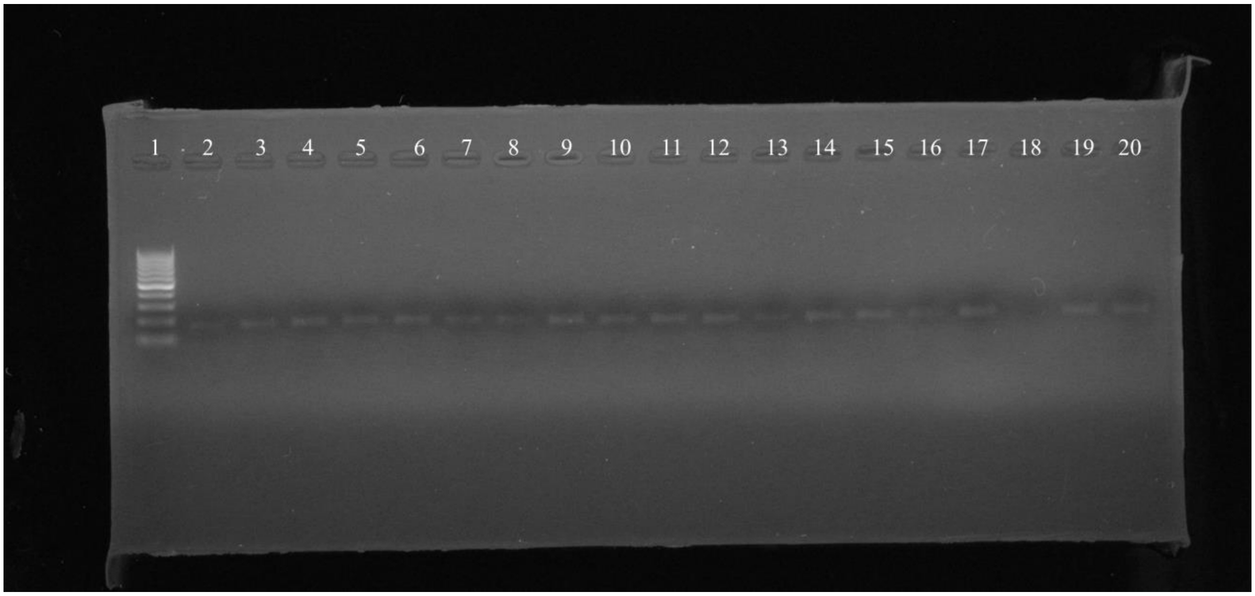

Figure S2. Original image a) Recombinant plasmid construction. Lane 1: Recombinant uncut plasmid, 2: Double digested (HindIII and NdeI) product of recombinant plasmid, 3 ladder. b) Showing the un-induced, induced BL21 cells at 37°C and purified protein. Lane 1: Purified protein, 2: ladder (in kDa), 3: Uninduced cells and 4: Induced cells.

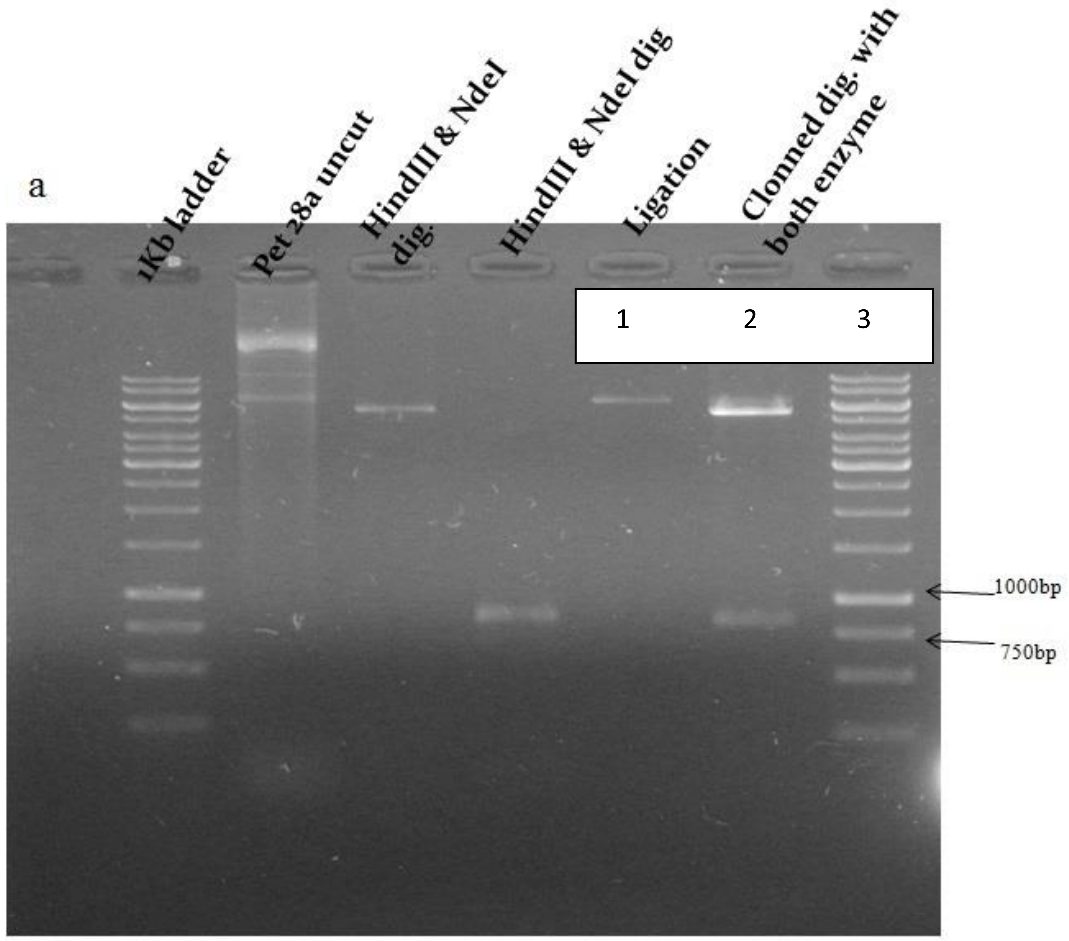

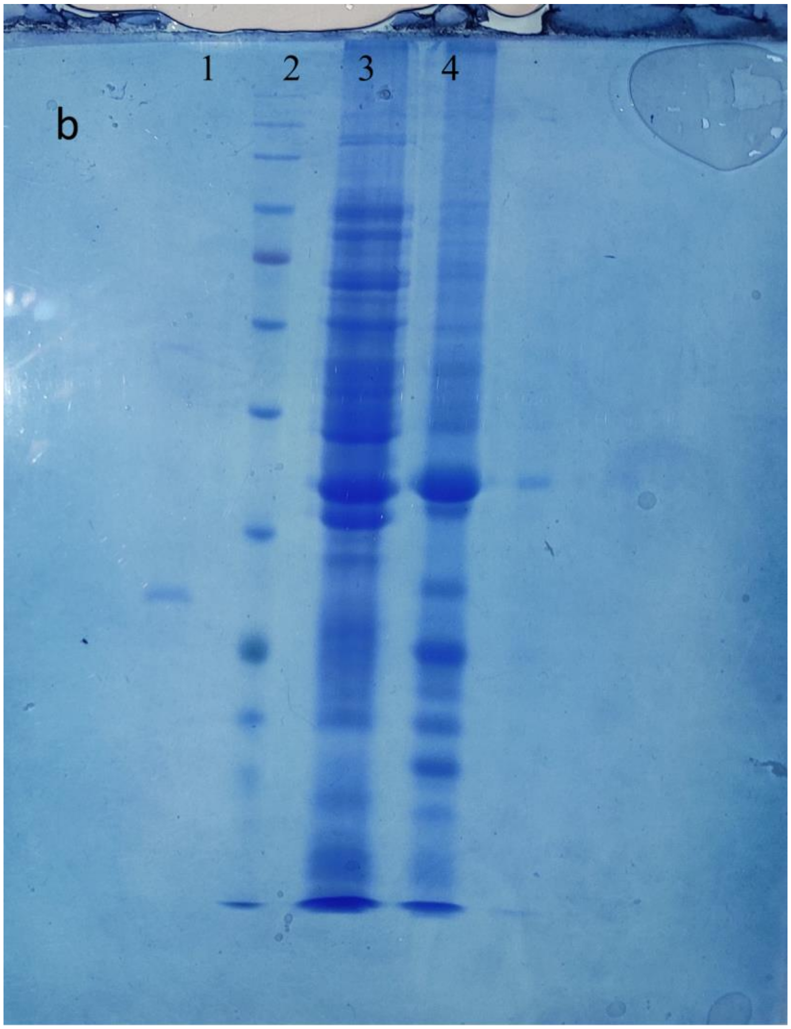
b
1
2
3
4

Figure S3: CdtB clonned vector, purified using gel extraction kit was sequenced using universal T7 promoter and T7 terminator primers in order to check the insertion of correct sequence into the vector. The sequencing confirmed the insertion of correct sequence into the pET28a vector

T7 promoter

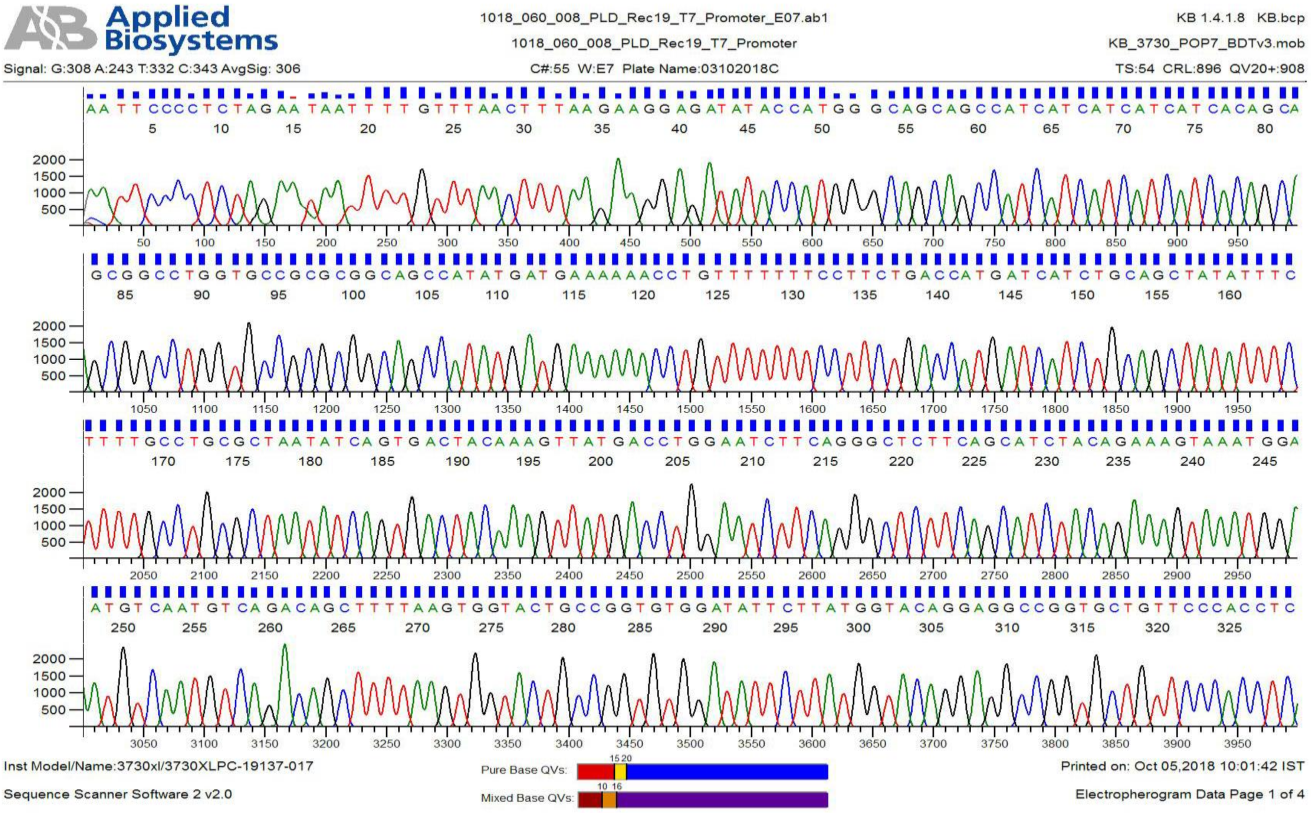

Applied Biosystems

1018_060_008_PLD_Rec19_T7_Promoter_E07.ab1
1018_060_008_PLD_Rec19_T7_Promoter
C#:55 W:E7 Plate Name:03102018C

KB 1.4.1.8 KB.bcp
KB_3730_POP7_BDTv3.mob
TS:54 CRL:896 QV20+:908

Signal: G:308 A:243 T:332 C:343 AvgSig: 306

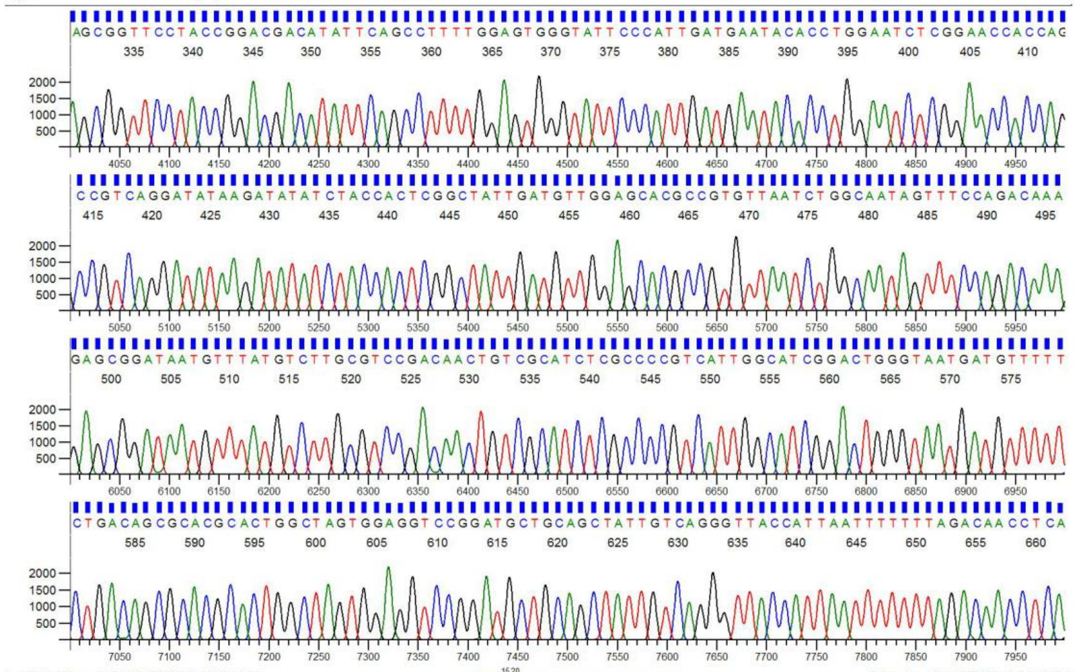

Inst Model/Name:3730xl/3730XLPC-19137-017
Sequence Scanner Software 2 v2.0

Pure Base QVs: 15 20
Mixed Base QVs: 10 16

Printed on: Oct 05,2018 10:01:42 IST
Electropherogram Data Page 2 of 4

Applied Biosystems

1018_060_008_PLD_Rec19_T7_Promoter_E07.ab1
1018_060_008_PLD_Rec19_T7_Promoter
C#:55 W:E7 Plate Name:03102018C

KB 1.4.1.8 KB.bcp
KB_3730_POP7_BDTv3.mob
TS:54 CRL:896 QV20+:908

Signal: G:308 A:243 T:332 C:343 AvgSig: 306

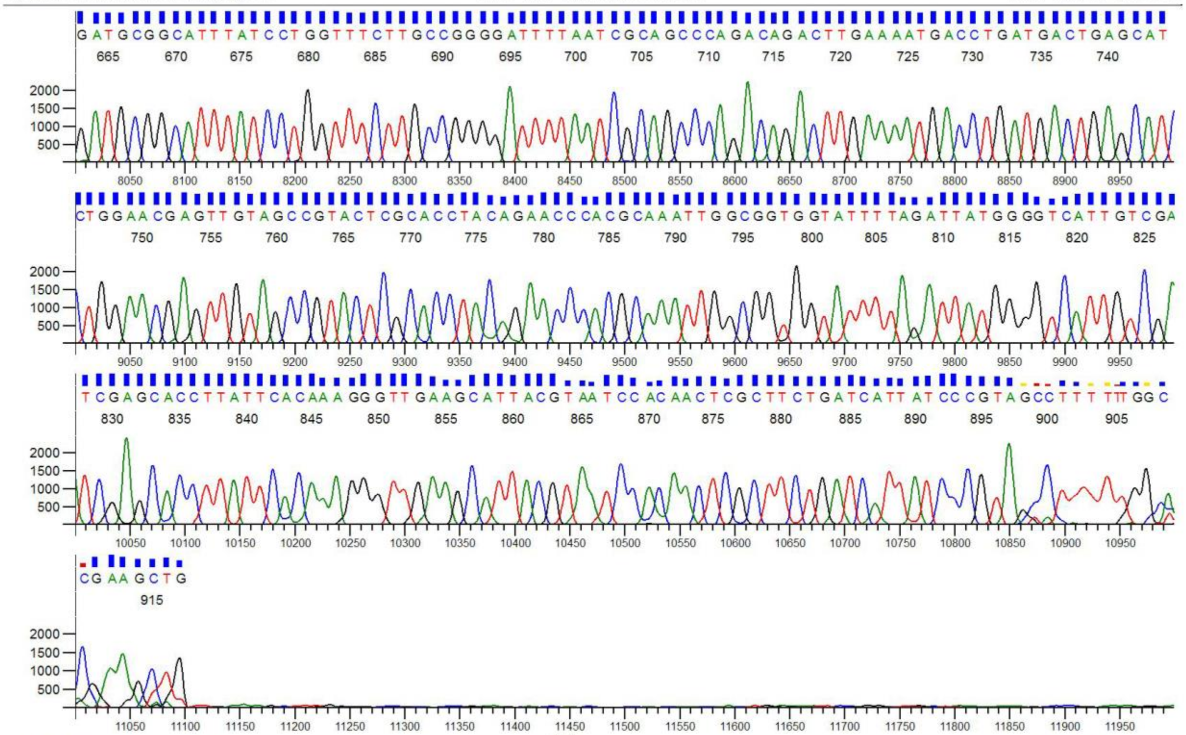

Inst Model/Name:3730xl/3730XLPC-19137-017
Sequence Scanner Software 2 v2.0

Pure Base QVs: 15 20
Mixed Base QVs: 10 16

Printed on: Oct 05,2018 10:01:42 IST
Electropherogram Data Page 3 of 4

T7 terminator

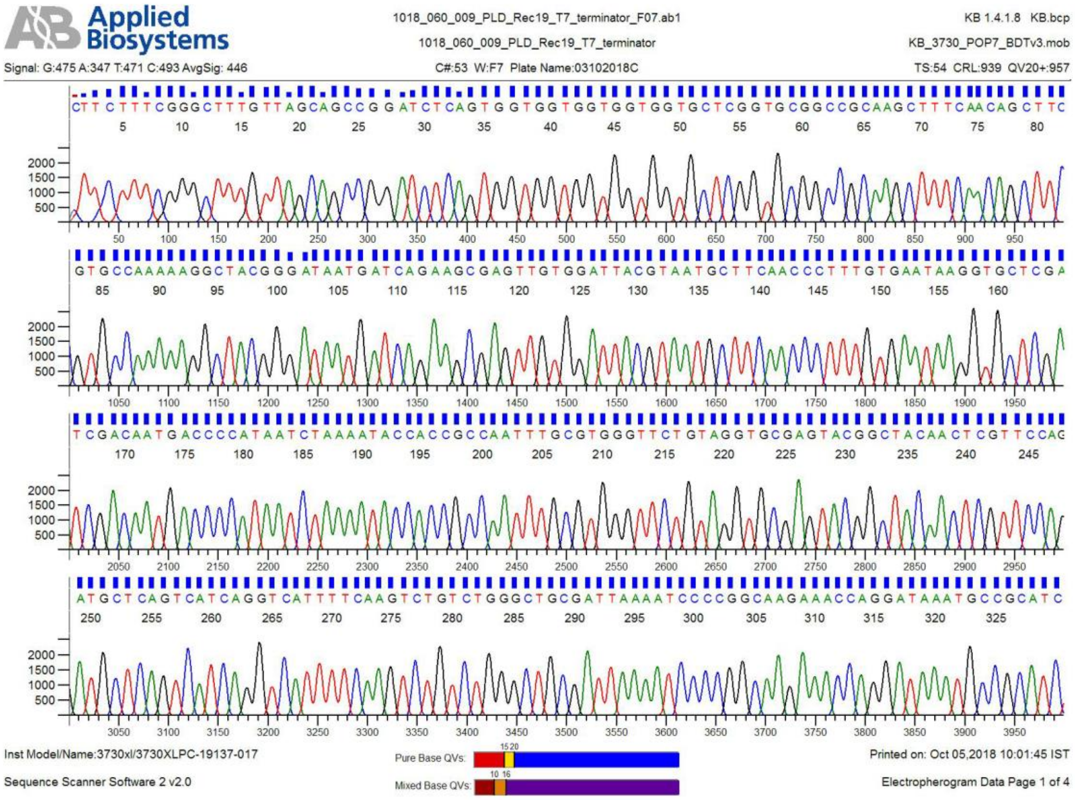

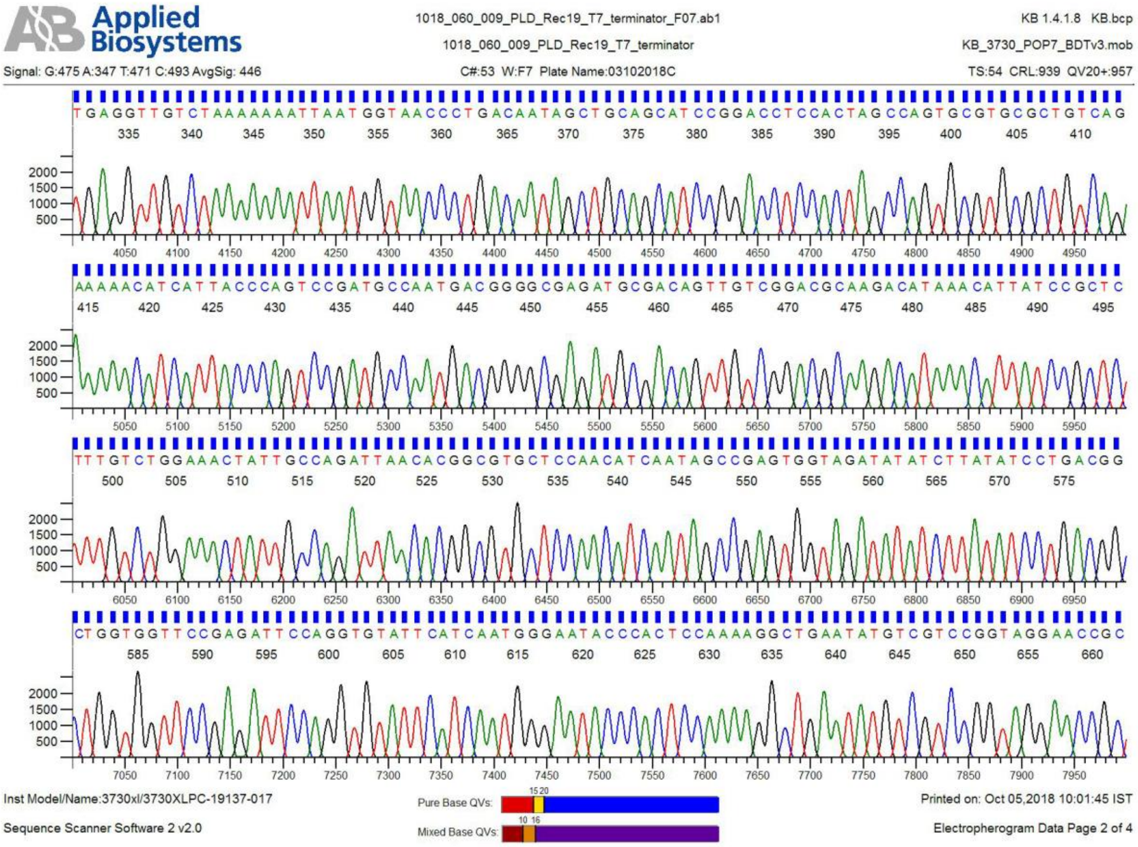

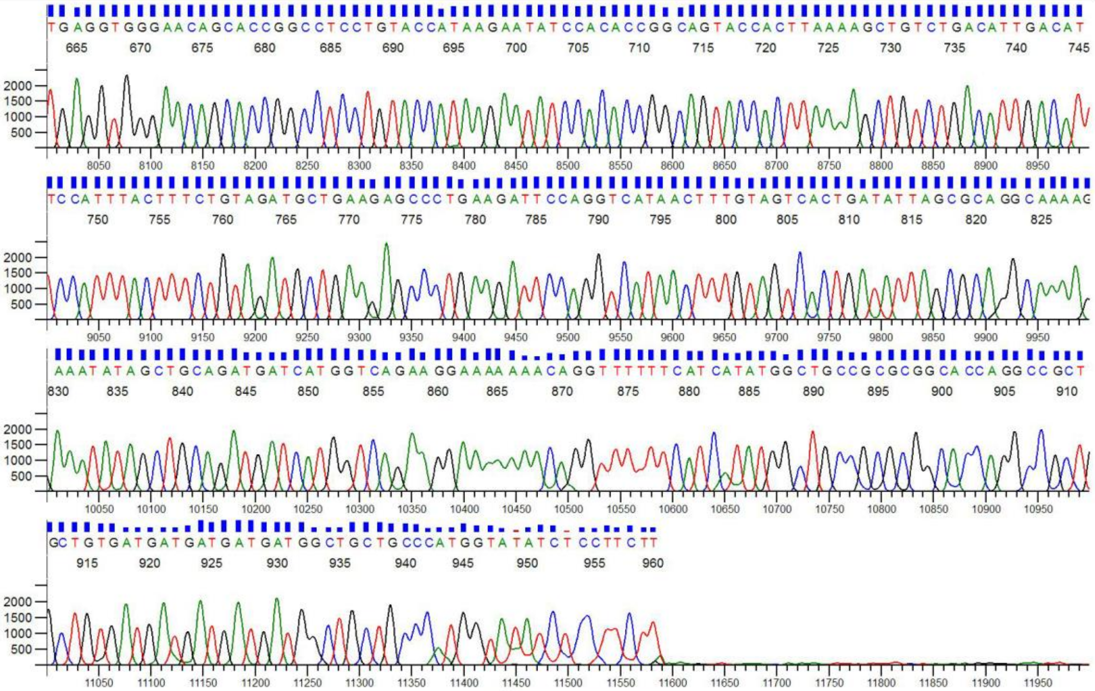
TGAGGTGGGAACAGCACCGGCCTCCTGTACCATAAGAATATCCACACCGGCAGTACCACTTAAAAGCTGTCTGACATTGACAT
665 670 675 680 685 690 695 700 705 710 715 720 725 730 735 740 745
TCCATTTACTTTCTGTAGATGCTGAAGAGCCCTGAAGATTCCAGGTCATAACTTTGTAGTCACTGATATTAGCGCAGGCAAAAG
750 755 760 765 770 775 780 785 790 795 800 805 810 815 820 825
AAATATAGCTGCAGATGATCATGGTCAGAAGGAAAAAAACAGGTTTTTCATCATATGGCTGCCGCGCGGCACCAGGCCGCT
830 835 840 845 850 855 860 865 870 875 880 885 890 895 900 905 910
GCTGTGATGATGATGATGATGGCTGCTGCCCATGGTATATCTCCTTCTT
915 920 925 930 935 940 945 950 955 960

Figure S4: a, b and c are the pictures of 1% gel and d, e & f are of 2% agarose gel. Both set of experiments performed thrice.

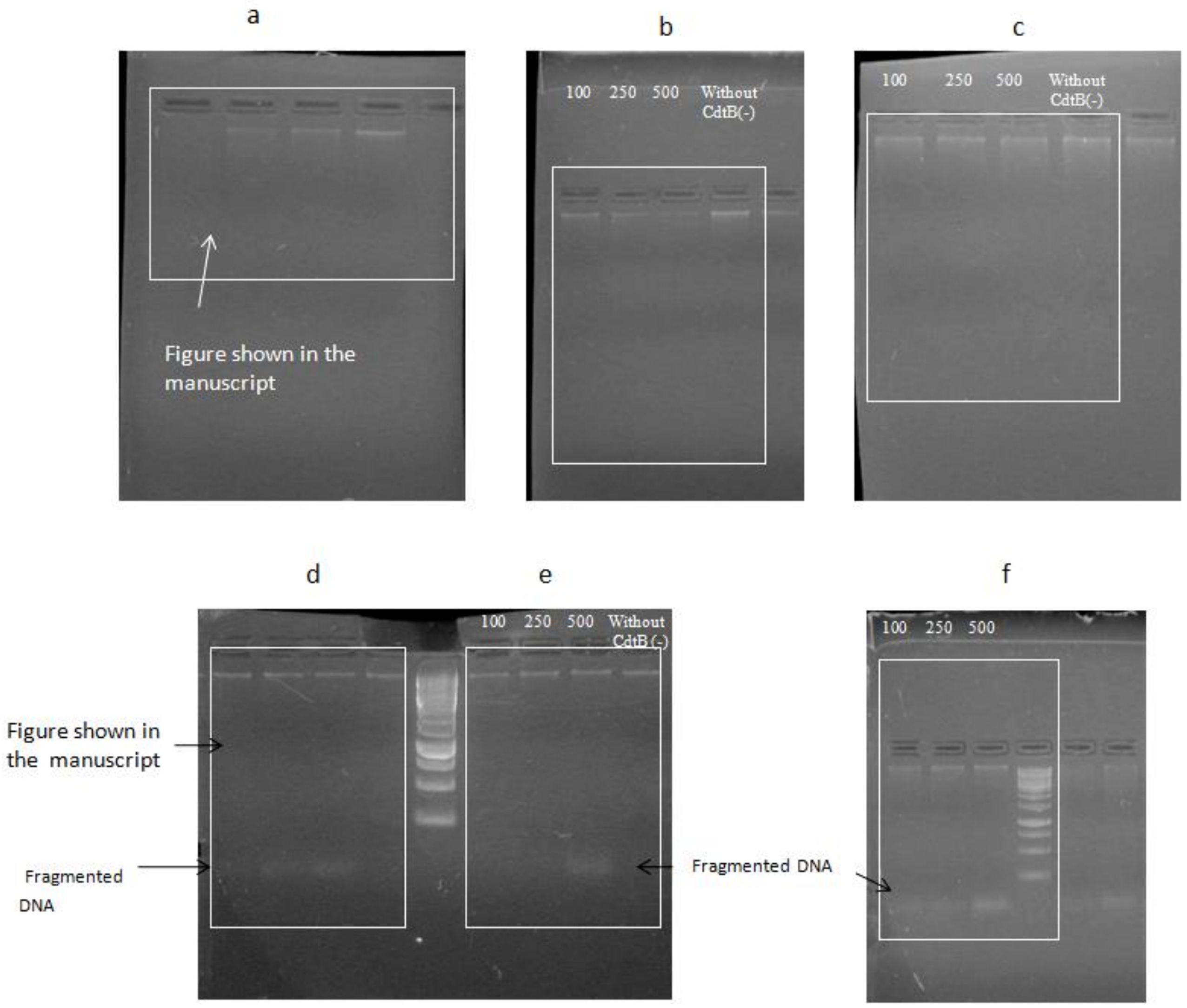

Figure-S5 No, significant morphological alterations were not observed in: a) RAW 264.7 (Mouse) and b) Caco-2 (Human) cell lines at different concentrations (1, 10 and 100 µg/ml of protein, 200 X magnifications).

a) RAW 264.7

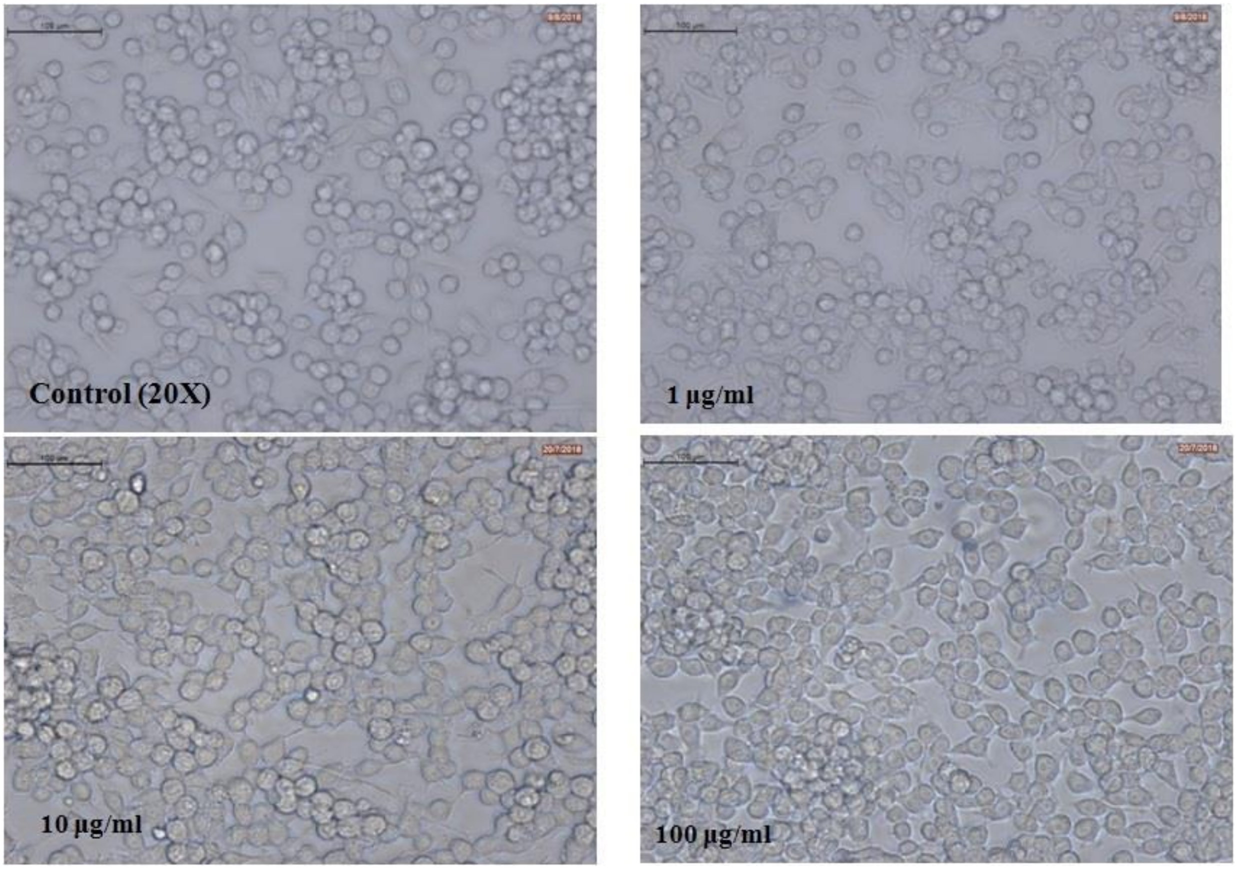

b) Caco-2

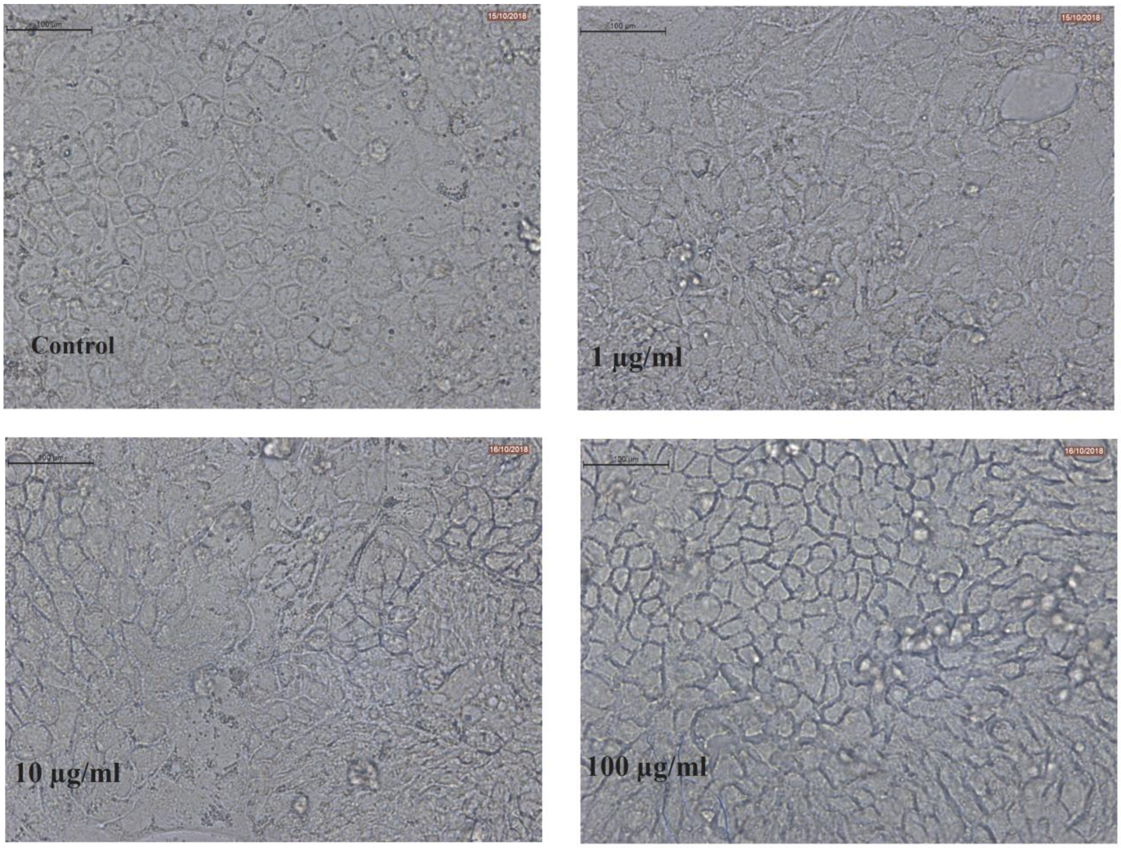
15/10/2018
Control
15/10/2018
1 µg/ml
16/10/2018
10 µg/ml
16/10/2018
100 µg/ml

Figure S6 . Cell cycle analysis of Caco-2 cells. fig. a) represents cells in different phases of cell cycle, b(i) representative figure of control cells and b(ii), (iii) & (iv) represent cells treated with 1, 10, &100 μg/ml concentrations of protein, respectively. No, significant difference was observed in control and treated cells.

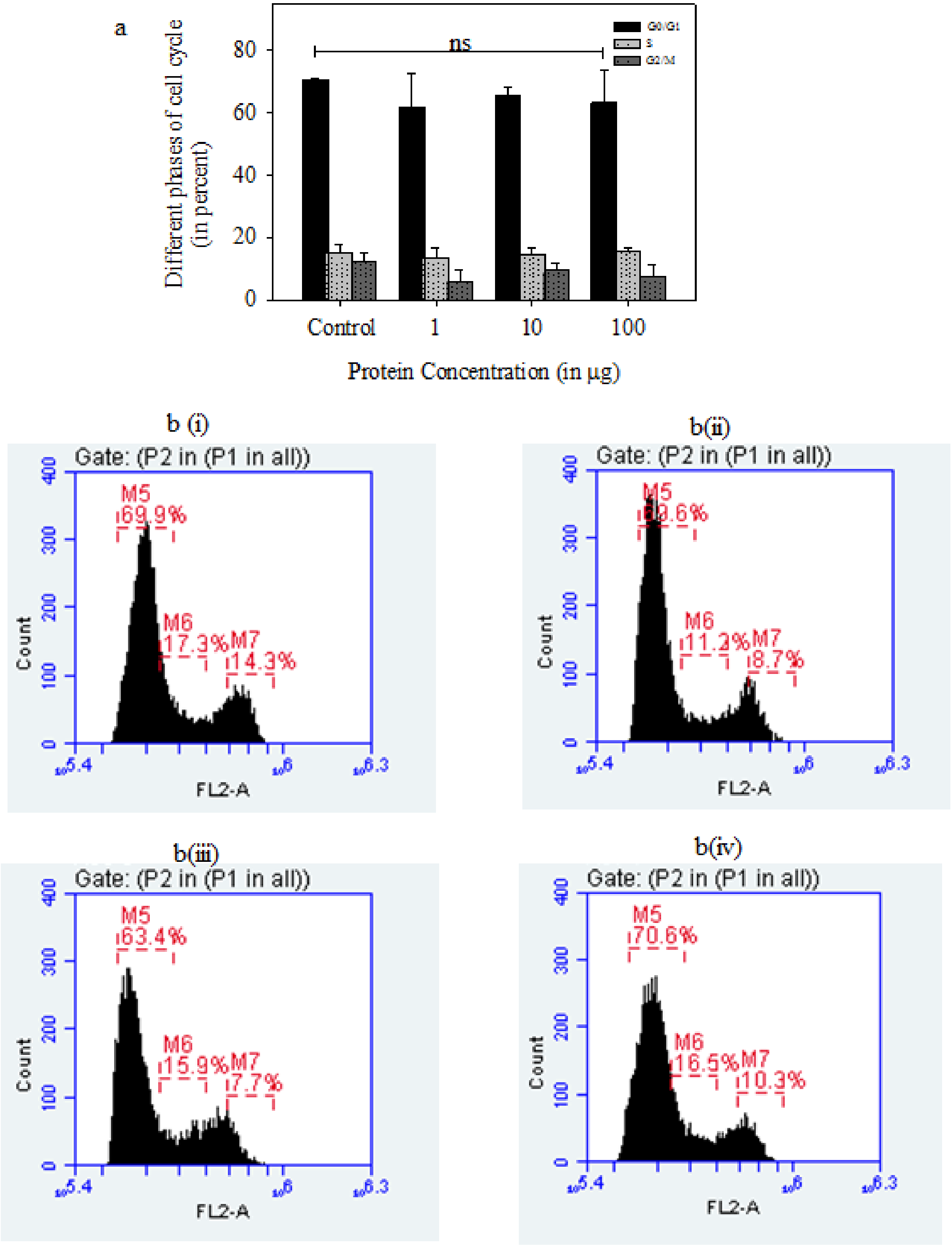

Figure S7. Original image, Western blot of two serum samples of mice (sera dilution used 1: 500) after the second booster (23rd day) showed evident blot, indicating the generation of anti-CdtB antibodies**.**

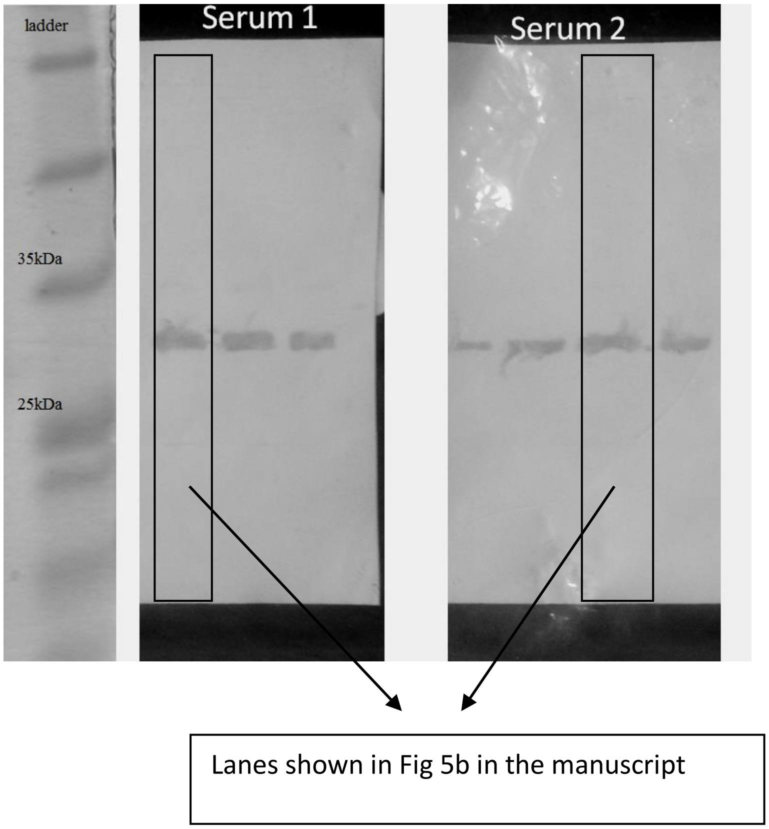

Figure S8: Histopathology of the spleen (40X) and liver (100X), high quality images**. Figure a, c, e, g are of spleen from** unimmunized-uninfected, unimmunized-infected (20 h), immunized-infected (20 h) and 7th day, respectively. **Figure b, d, f, h are of Liver from** unimmunized-uninfected, unimmunized-infected (20 h), immunized-infected (20 h) and 7th day, respectively. Description of image has been given in the manuscript.

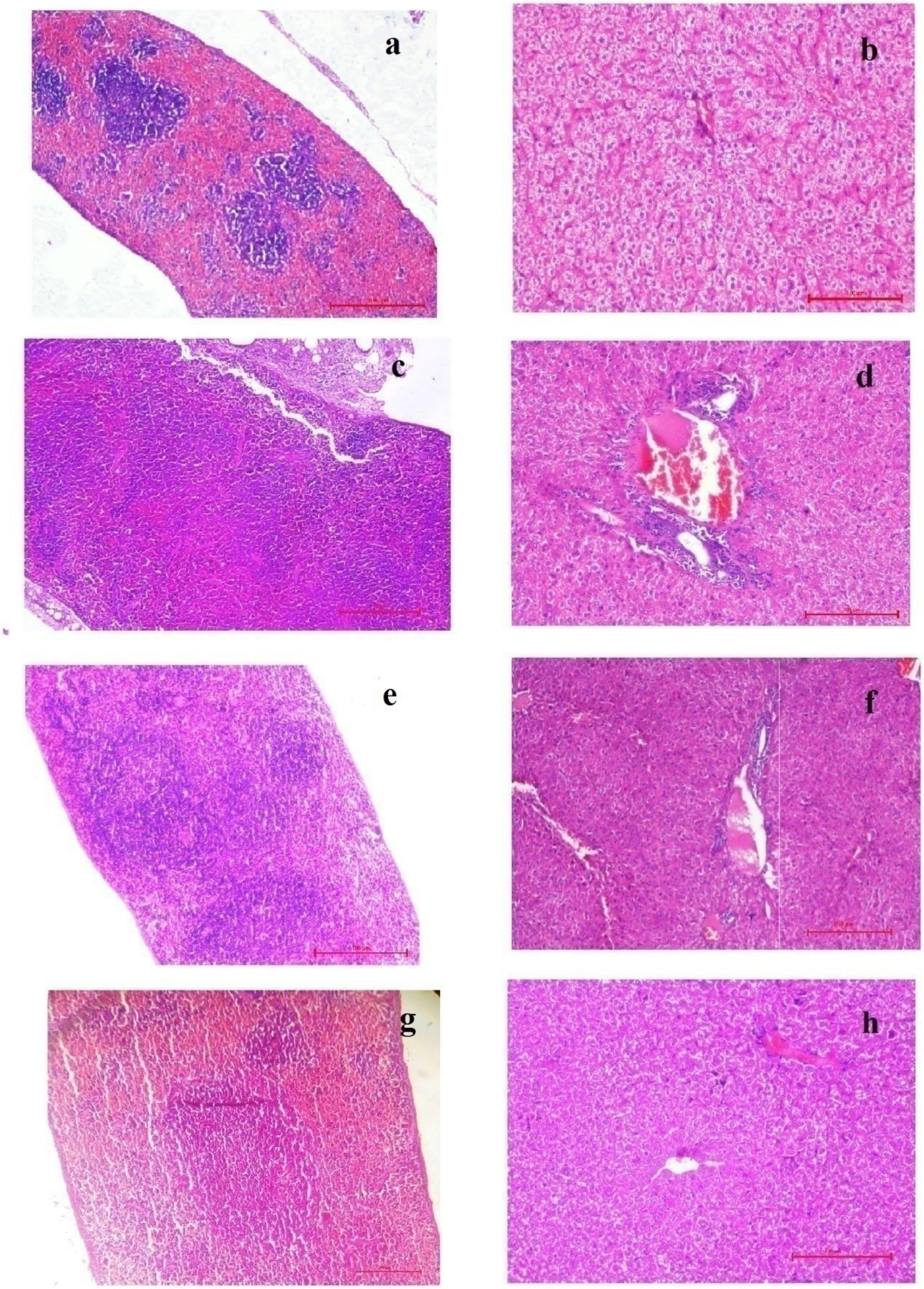

Supplement: Supplementary file 1 — supplementary information [file 41598_2019_54690_MOESM1_ESM.pdf]
